# PolyQ-expanded proteins impair cellular proteostasis of ataxin-3 through sequestering the co-chaperone HSJ1 into aggregates

**DOI:** 10.1038/s41598-021-87382-w

**Published:** 2021-04-09

**Authors:** Hong-Wei Yue, Jun-Ye Hong, Shu-Xian Zhang, Lei-Lei Jiang, Hong-Yu Hu

**Affiliations:** 1 State Key Laboratory of Molecular Biology, Shanghai Institute of Biochemistry and Cell Biology, Center for Excellence in Molecular Cell Science, Chinese Academy of Sciences, Shanghai, 200031 People’s Republic of China; 2 University of Chinese Academy of Sciences, Beijing, 100049 People’s Republic of China

**Keywords:** Protein aggregation, Mechanisms of disease

## Abstract

Polyglutamine (polyQ) expansion of proteins can trigger protein misfolding and amyloid-like aggregation, which thus lead to severe cytotoxicities and even the respective neurodegenerative diseases. However, why polyQ aggregation is toxic to cells is not fully elucidated. Here, we took the fragments of polyQ-expanded (PQE) ataxin-7 (Atx7) and huntingtin (Htt) as models to investigate the effect of polyQ aggregates on the cellular proteostasis of endogenous ataxin-3 (Atx3), a protein that frequently appears in diverse inclusion bodies. We found that PQE Atx7 and Htt impair the cellular proteostasis of Atx3 by reducing its soluble as well as total Atx3 level but enhancing formation of the aggregates. Expression of these polyQ proteins promotes proteasomal degradation of endogenous Atx3 and accumulation of its aggregated form. Then we verified that the co-chaperone HSJ1 is an essential factor that orchestrates the balance of cellular proteostasis of Atx3; and further discovered that the polyQ proteins can sequester HSJ1 into aggregates or inclusions in a UIM domain-dependent manner. Thereby, the impairment of Atx3 proteostasis may be attributed to the sequestration and functional loss of cellular HSJ1. This study deciphers a potential mechanism underlying how PQE protein triggers proteinopathies, and also provides additional evidence in supporting the hijacking hypothesis that sequestration of cellular interacting partners by protein aggregates leads to cytotoxicity or neurodegeneration.

## Introduction

Polyglutamine (polyQ) diseases are one group of neurodegenerative disorders typically characterized by the aberrant expansion of *CAG* repeats that encodes a polyQ tract in the pathogenic protein^1,2^. The existing polyQ tract that extends over a certain threshold will become an amyloid core and thus trigger protein misfolding and aggregation^3^. It is well-known that protein aggregation is a pathological hallmark shared among diverse neurodegenerative diseases^4–8^. However, why amyloid-like aggregation of polyQ proteins is toxic to cells, especially to neurons, remains only partially understood. As each polyQ disease is caused by one definitive pathogenic protein that possesses a clear amyloidogenic core, the expanded polyQ tract, it becomes an excellent model for studying the patho-mechanism of protein aggregation-related diseases^9^. The pathogenic polyQ-expanded (PQE) proteins can be cleaved to generate small fragments by intracellular proteases^10^. So far, different N-terminal fragments of PQE huntingtin (Htt)^11–13^ and ataxin-7 (Atx7)^14–16^ and the C-terminal fragments of pathogenic ataxin-3 (Atx3)^17–20^ have been identified and characterized. These small fragments with expanded polyQ tracts are sufficient to cause cytotoxicities or produce disease phenotypes. Therefore, the small fragments with polyQ tracts have been applied in many cellular research systems and animal models to elucidate the cytotoxicities and pathologies of polyQ diseases^21,22^. In our previous studies, we took several fragments of PQE Htt^23,24^, Atx3^24,25^ and Atx7^26^ as models to address such a general question why polyQ aggregates are toxic to cells or neurons.

Recently, oligomers and aggregates generated during protein misfolding are recognized as two major toxic species^27^. The soluble oligomers and deposited aggregates seem to exhibit cytotoxicities through diverse mechanisms. Protein oligomers expose their hydrophobic amino-acid residues to form sticky surfaces that are reported to aberrantly interact with key cellular factors^28^ or disrupt phosphorlipid bilayers^29,30^. Protein aggregates deposited in cells usually form inclusion bodies, the common structures that can be visualized in different neurodegenerative diseases^31–33^. Accumulating evidence supports that protein aggregates or inclusions are toxic to cells by sequestering or hijacking critical cellular components, including chaperones and co-chaperones^7,34–36^, ubiquitin (Ub)-related proteins^24,37,38^, and other specifically interacting partners^25,26^. These sequestration effects may cause loss-of-function of the hijacked components and thus lead to their cellular dysfunction and cytotoxicity. For example, polyQ aggregates as reported can sequester the HSP40 chaperone Sis1p, interfere with the normal function of hijacked Sis1p, and consequently result in impairment of the cellular proteostasis^39^. Thus, the diversity of composition in protein aggregates or inclusions may be at least partially explained by the sequestration effect^40^. Diverse proteins appeared in the polyQ inclusions are potentially related to the cytotoxicity and pathological progression of the polyQ diseases.

Ataxin-3 (Atx3) is one of the deubiquitinating enzymes reported to be involved in different protein inclusion bodies^41,42^. As a polyQ tract-containing protein, Atx3 may experience polyQ expansion that leads to protein aggregation and the spinal cerebellar ataxia (SCA3)^8,43^. It should be noted that wild-type (WT) Atx3 other than its pathogenic one has also been identified in the polyQ inclusions, suggesting that the WT Atx3 protein may be sequestered into the aggregates in cells^17,44^. Our previous study unraveled that the proteasomal degradation of endogenous Atx3 is orchestrated delicately by HSJ1 (DNAJB2), a co-chaperone of HSP70^45^. HSJ1 (mainly HSJ1a and HSJ1b isoforms) belongs to a member of the DNAJ family proteins that are defined by the J domain (JD), and regulates the function of HSP70 chaperones^46^. HSP70 and its co-chaperones cooperate closely with the protein degradation machineries in a concerted proteostasis network^47^.

We hypothesized that the involvement of endogenous Atx3 in the polyQ inclusions could account for the cytotoxic effects of the pathogenic PQE proteins. To address this issue, we applied the N-terminal fragments of PQE Atx7 and Htt proteins to examine the effect of polyQ aggregates on the cellular proteostasis of Atx3. We found that these PQE proteins promote proteasomal degradation of endogenous Atx3 and enhance its formation of aggregates. We also confirmed our previous finding that HSJ1 functions as a dual modulator of Atx3 in degradation and stability^45^. Besides, the impairment of cellular proteostasis of Atx3 may be attributed to the sequestration of HSJ1 and its functional loss by the PQE proteins. This study provides some clues to understanding of the mechanism underlying why PQE protein is toxic to cells.

## Results

### PQE Atx7 and Htt reduce the soluble fraction of endogenous Atx3 but increase its insoluble aggregates

**Figure 1:**
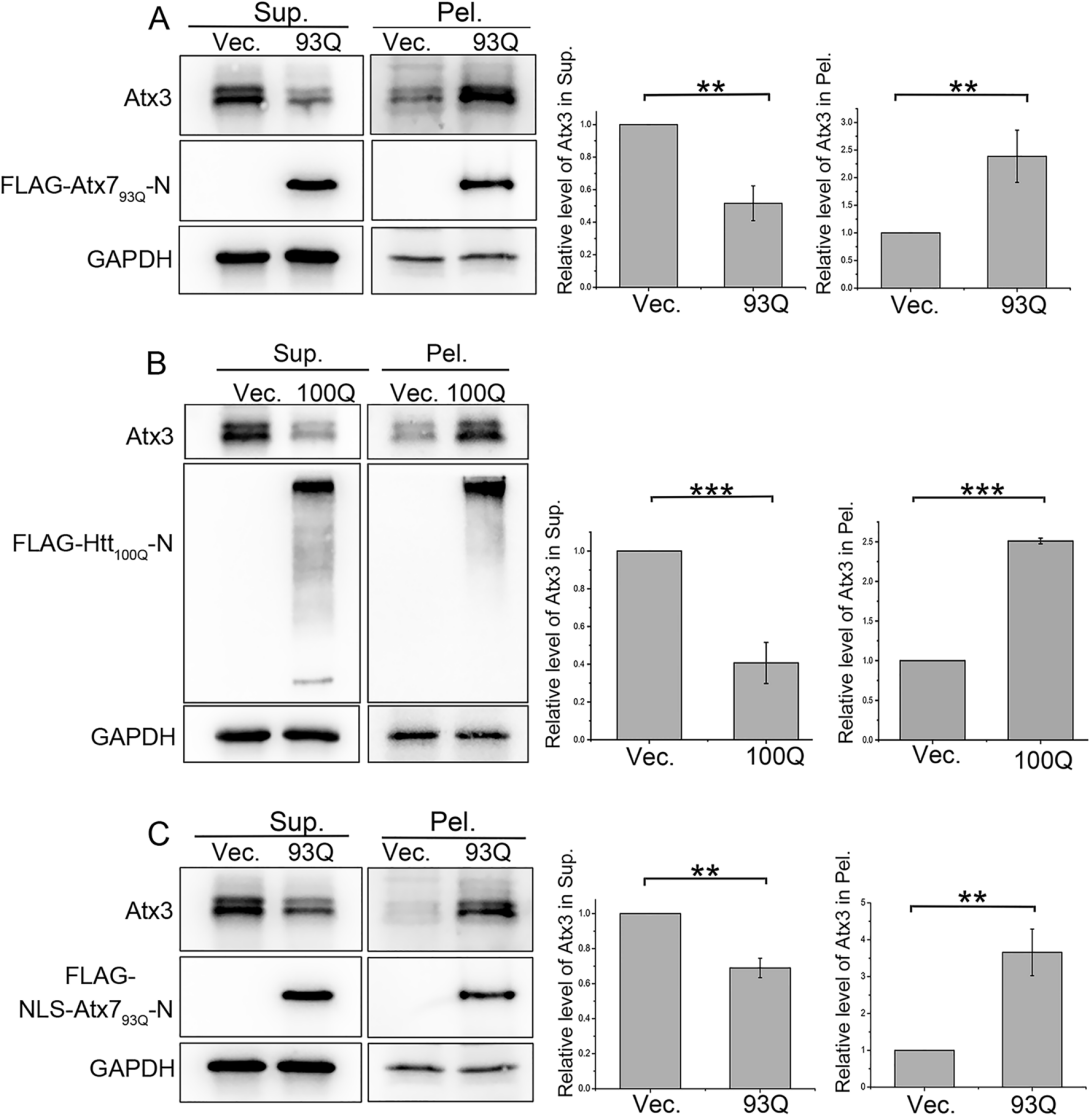
PQE Atx7 and Htt reduce the soluble fraction of intracellular Atx3 but increase the insoluble. (**A**) Effects of cytoplasm-localized Atx7_93Q_-N172 on the supernatant and pellet fractions of Atx3. (**B**) As in (**A**), cytoplasm-localized Htt_100Q_-N90. (**C**) As in (**A**), nucleus-localized NLS-Atx7_93Q_-N172. HEK 293T cells were transfected with each indicated plasmid and the cell lysates were subjected to supernatant/pellet fractionation and Western blotting analysis. Indicated proteins were detected by using anti-FLAG, anti-Atx3 and anti-GAPDH antibodies. The two main bands indicate different isoforms of endogenous Atx3, Atx3-I and Atx3-II. Sup., supernatant; Pel., pellet. The gray values of Atx3 protein bands from three independent experiments were employed for statistical analysis. Data are shown as Means ± SEM (n = 3). **p < 0.01; ***p < 0.001.

**Figure 2:**
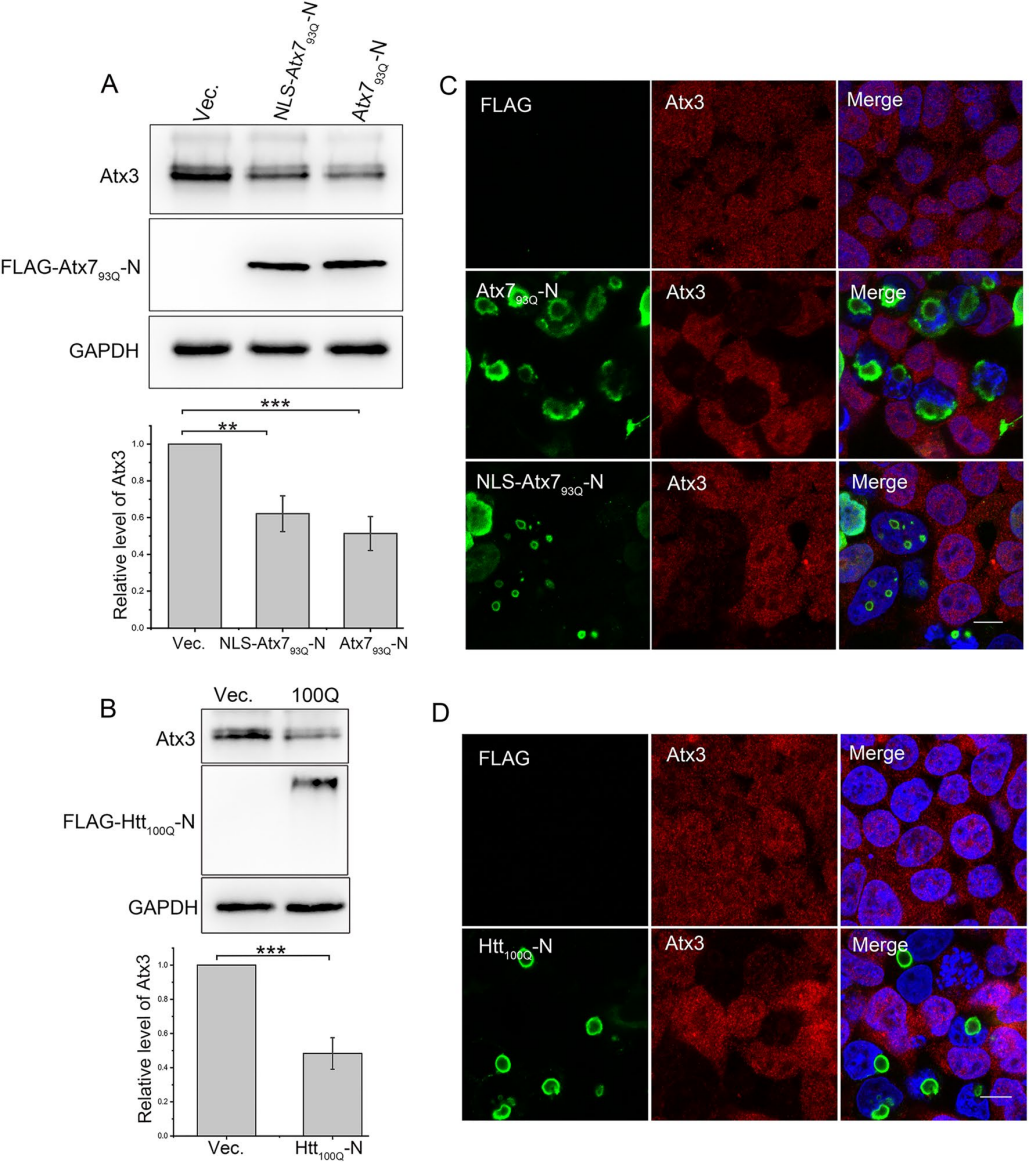
PQE Atx7 and Htt reduce the overall protein level of intracellular Atx3. (**A**) Effect of Atx7_93Q_-N172 or NLS-Atx7_93Q_-N172 on the overall level of Atx3. (**B**) Effect of Htt_100Q_-N90 on the overall level of Atx3. HEK 293T cells were transfected with each indicated plasmid and the total-protein samples were prepared and subjected to Western blotting. Indicated proteins were detected by using anti-FLAG, anti-Atx3 and anti-GAPDH antibodies. Data are shown as Means ± SEM (n = 3). **p < 0.01; ***p < 0.001. (**C**) Immunofluorescence imaging showing that the cytoplasmic or nuclear Atx7_93Q_-N172 inclusions cause fluorescence weakening of the endogenous Atx3. (D) As in (C), Htt_100Q_-N90. HEK 293T cells were transfected with each indicated plasmid and then subjected to immunofluorescence staining and confocal microscopic imaging. PolyQ proteins were stained with anti-FLAG antibody (green), Atx3 was stained with anti-Atx3 antibody (red), and nuclei were stained with Hoechst (blue). Scale bar = 10 μm.

 Atx3 is a polyQ tract-containing protein that may co-aggregate with other PQE proteins and form amyloid-like inclusions in cells^17,44^. To examine the possible co-aggregation effect of the polyQ proteins on the cellular Atx3 level, we firstly applied the N-terminal fragment of PQE Atx7 (Atx7_93Q_-N172) and performed supernatant/pellet fractionation experiment in HEK 293T cells. As shown in the result, expression of Atx7_93Q_-N172 caused a remarkable decline of soluble Atx3 in the supernatant fraction; whereas it increased the insoluble aggregates appeared in the pellet (Fig. 1A). We then verified this effect in an N-terminal fragment of PQE Htt (Htt_100Q_-N90). It showed a similar effect with Atx7_93Q_-N172 on the endogenous Atx3 levels both in soluble and insoluble fractions (Fig. 1B). Considering that both Atx7_93Q_-N172 and Htt_100Q_-N90 form cytoplasmic aggregates or inclusions, we further determined to clarify whether the nucleus-localized form of Atx7_93Q_-N172 (NLS-Atx7_93Q_-N172) similarly influence the cellular Atx3 levels. The nucleus-localized construct formed nuclear inclusions (see Fig. 2C); it could also reduce the soluble Atx3 level but increase its insoluble fraction (Fig. 1C). All these results demonstrate that the PQE proteins can reduce the soluble Atx3 level but enhance formation of the aggregates, regardless of their polyQ-protein types or cellular localization.

### PQE Atx7 and Htt reduce the overall level of intracellular Atx3

 As a remarkable decline of the soluble Atx3 fraction was observed in the supernatant, we wondered whether this reduction was due to its conversion into insoluble fraction or reflected a decrease in the overall protein level. We thus extracted total intracellular protein of Atx3 by using the lysis buffer containing 8 M urea that may completely dissolve the aggregated form of Atx3, and then detected the overall protein level of endogenous Atx3 by Western blotting. The results showed that Atx7_93Q_-N172, whether localized in cytoplasm or nucleus, caused a significant decrease in the total protein level of Atx3 (Fig. 2A). Similarly, Htt_100Q_-N90 could also reduce the total Atx3 level (Fig. 2B). Besides, as visualized by confocal microscopy, the immunofluorescence intensity of endogenous Atx3 dropped down obviously upon formation of the Atx7_93Q_-N172 inclusions (Fig. 2C). Consistently, we also observed a decrease of the Atx3 fluorescence in the cells with formation of the Htt_100Q_-N90 inclusions (Fig. 2D). So, we proposed that the PQE proteins reduce the soluble protein of endogenous Atx3 and thus cause decrease of the overall protein level, albeit the insoluble Atx3 aggregates increase substantially.

To examine whether the decrease of Atx3 level was caused by mRNA variation, we performed quantitative RT-PCR assay for evaluating the mRNA level changes upon exogenous expression of PQE proteins. The qPCR data showed that the mRNA levels of Atx3 remained almost unchanged when the Atx7_93Q_-N172 or Htt_100Q_-N90 protein was exogenously over-expressed in cells (Supplemental Fig. 1), suggesting that overexpression of the PQE proteins has little effect on the mRNA level of Atx3. This result excludes the possibility that the decrease of Atx3 level is due to its transcriptional alteration.

### PQE Atx7 and Htt promote proteasomal degradation of intracellular Atx3

 As well acknowledged, cellular proteostasis is finely regulated by an integrated network of chaperones and protein degradation system^48–51^. However, the misfolded proteins that escape surveillance of the degradation machineries would form insoluble aggregates deposited in cells^27^. Therefore, we speculate that, upon expression of the PQE proteins, the cellular proteostasis of Atx3 is somehow disrupted, resulting in an increase of protein aggregates and a massive decline of soluble Atx3 that may be attributed to protein degradation.

**Figure 3:**
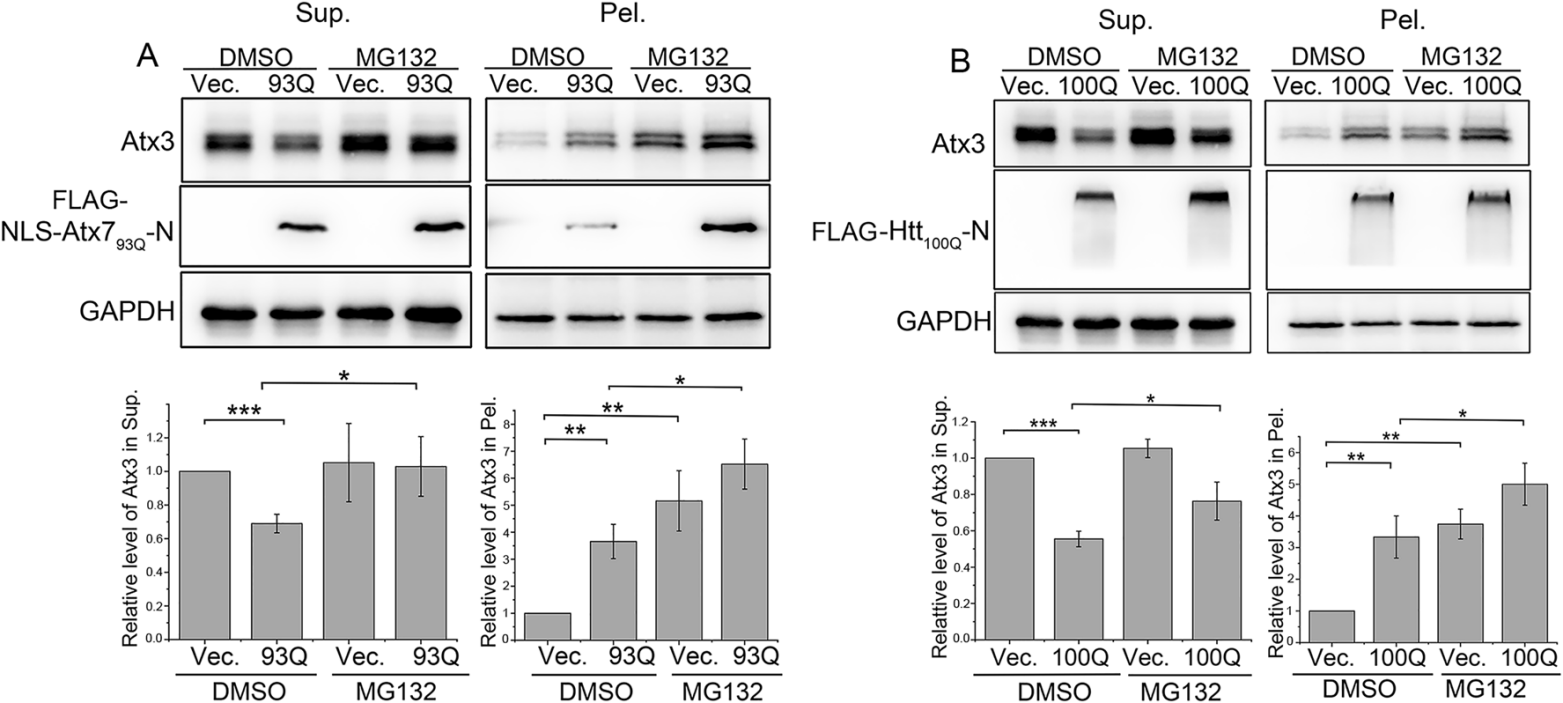
PQE Atx7 and Htt cause the reduction of soluble Atx3 through promoting proteasomal degradation. (**A**) Inhibition of proteasome suppresses the decline of soluble Atx3 caused by NLS-Atx7_93Q_-N172. (B) As in (A), by Htt_100Q_-N90. HEK 293T cells were transfected with each indicated plasmid and treated with MG132 (10 μM) for 12 h before harvest. DMSO was set as a control. The cell lysates were subjected to supernatant/pellet fractionation and Western blotting. Indicated proteins were detected by using anti-FLAG, anti-Atx3 and anti-GAPDH antibodies. Sup., supernatant; Pel., pellet. Data are shown as Means ± SEM (n = 3). *p < 0.05; **p < 0.01; ***p < 0.001.

To verify the role of proteasomal degradation pathway in reduction of the Atx3 level triggered by the PQE proteins, we examined the effect of the proteasome inhibitor MG132 on the degradation of endogenous Atx3. The results showed that, NLS-Atx7_93Q_-N172 and Htt_100Q_-N90 both caused reduction of the soluble Atx3 level, while MG132 treatment, to some extent, could reverse the decrease of Atx3 in the supernatant fraction (Fig. 3A, B). As expected, MG132 treatment aggravated the increase of the aggregated form of Atx3 caused by the PQE proteins (Fig. 3A, B), which may be attributed to the inefficient degradation of Atx3 upon inhibition of proteasome. We then examined the possible role of autophagy pathway in Atx3 degradation by using chloroquine (CQ), a lysosomal degradation inhibitor. As a result, CQ treatment could up-regulate the LC3-II level remarkably (Supplemental Fig. 2A, C), indicating the effective inhibition of autophagic degradation. However, blocking of the autophagic degradation exhibited no significant influence on the decline of Atx3 caused by Atx7_93Q_-N172 or Htt_100Q_-N90 (Supplemental Fig. 2B, D). Similarly, we also observed the increase of the aggregated form of Atx3 in the pellet fraction upon inhibition of autophagy. As autophagy is widely recognized as an important pathway to scavenge protein aggregates^52,53^, it may exert a role in the clearance of Atx3 aggregates in cells. So, we conclude that the PQE proteins reduce the soluble form of intracellular Atx3 mainly through promoting proteasomal degradation.

### HSJ1a suppresses the degradation of intracellular Atx3 through its UIM domain

**Figure 4:**
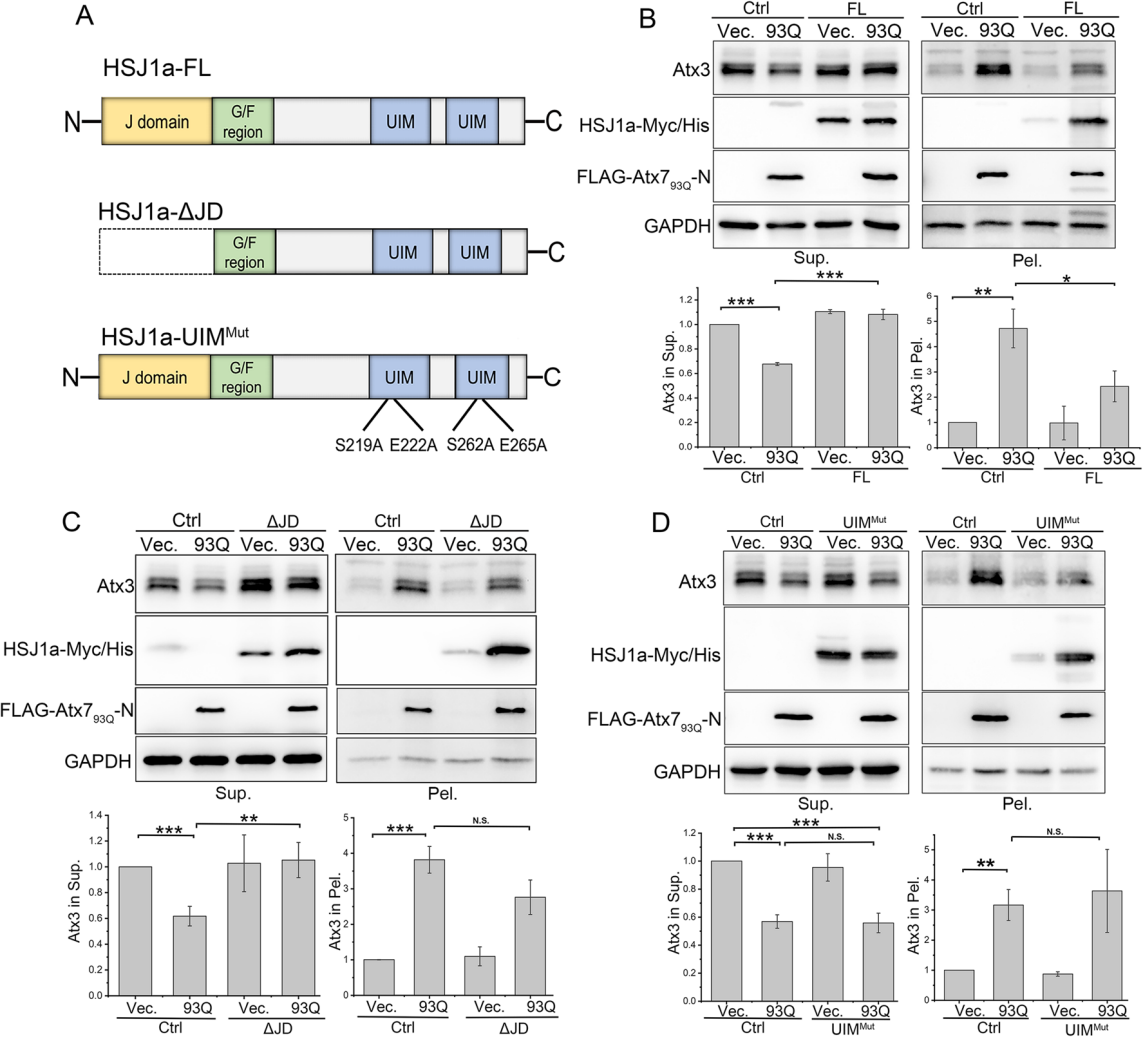
HSJ1a suppresses the degradation of Atx3 through its UIM domain. (**A**) Domain architectures of the HSJ1a protein and its mutants applied. (**B**) Overexpression of HSJ1a alleviates the decline of soluble Atx3 but still increases the aggregates caused by Atx7_93Q_-N172. (**C**) As in (**B**), HSJ1a-ΔJD. (**D**) Overexpression of HSJ1a-UIM^Mut^ fails to suppress the decline of soluble Atx3 caused by Atx7_93Q_-N172. HSJ1a-Myc/His or its mutants (HSJ1a-ΔJD and HSJ1a-UIM^Mut^) was co-expressed with Atx7_93Q_-N172 in HEK 293T cells. The pcDNA3.1-Myc/His plasmid was set as a control (Ctrl). The cell lysates were subjected to supernatant/pellet fractionation and Western blotting. Indicated proteins were detected by using anti-FLAG, anti-Myc, anti-Atx3 and anti-GAPDH antibodies. Sup., supernatant; Pel., pellet. Data are shown as Means ± SEM (n = 3). *p < 0.05; **p < 0.01; ***p < 0.001; N.S., no significance.

 We previously revealed that HSJ1a promotes proteasomal degradation of Atx3 through interacting with HSP70 mainly by the function of the J domain, while its UIM domain binds to the Ub chains conjugated in Atx3 and maintains its protein level^45^. Thus, we examined the effect of HSJ1a on the degradation of endogenous Atx3 triggered by the PQE proteins. We co-expressed full-length HSJ1a (HSJ1a-FL) (Fig. 4A) with Atx7_93Q_-N172 or NLS-Atx7_93Q_-N172 in HEK 293T cells and performed supernatant/pellet fractionation. The results showed that overexpression of HSJ1a-FL could both alleviate the reduction of soluble Atx3 in supernatant and the increase of aggregated Atx3 in pellet (Fig. 4B and Supplemental Fig. 3A). This implied that HSJ1a has an important function in modulating the cellular proteostasis of Atx3. As known, the J domain of HSJ1a exhibits a co-chaperone activity for promoting Atx3 degradation and consequently attenuating its aggregation^45^. We thus over-expressed the JD-deleted construct (HSJ1a-ΔJD) with Atx7_93Q_-N172 and found that, similar to HSJ1a-FL, HSJ1a-ΔJD was also able to reverse the reduction of soluble Atx3 caused by the PQE protein (Fig. 4C). Based on our previous finding that HSJ1a mainly suppresses the Atx3 degradation through its UIM domain^45^, we over-expressed the UIM-domain mutant of HSJ1a (HSJ1a-UIM^mut^) and revealed that the mutant failed to reverse the reduction of soluble Atx3 (Fig. 4D and Supplemental Fig. 3B). Together, these data reaffirm that HSJ1a suppresses the degradation of Atx3 through the UIM domain, and thus suggest that it is able to reverse the reduction of soluble Atx3 caused by the PQE proteins.

### PQE Atx7 and Htt sequester HSJ1a into aggregates

**Figure 5:**
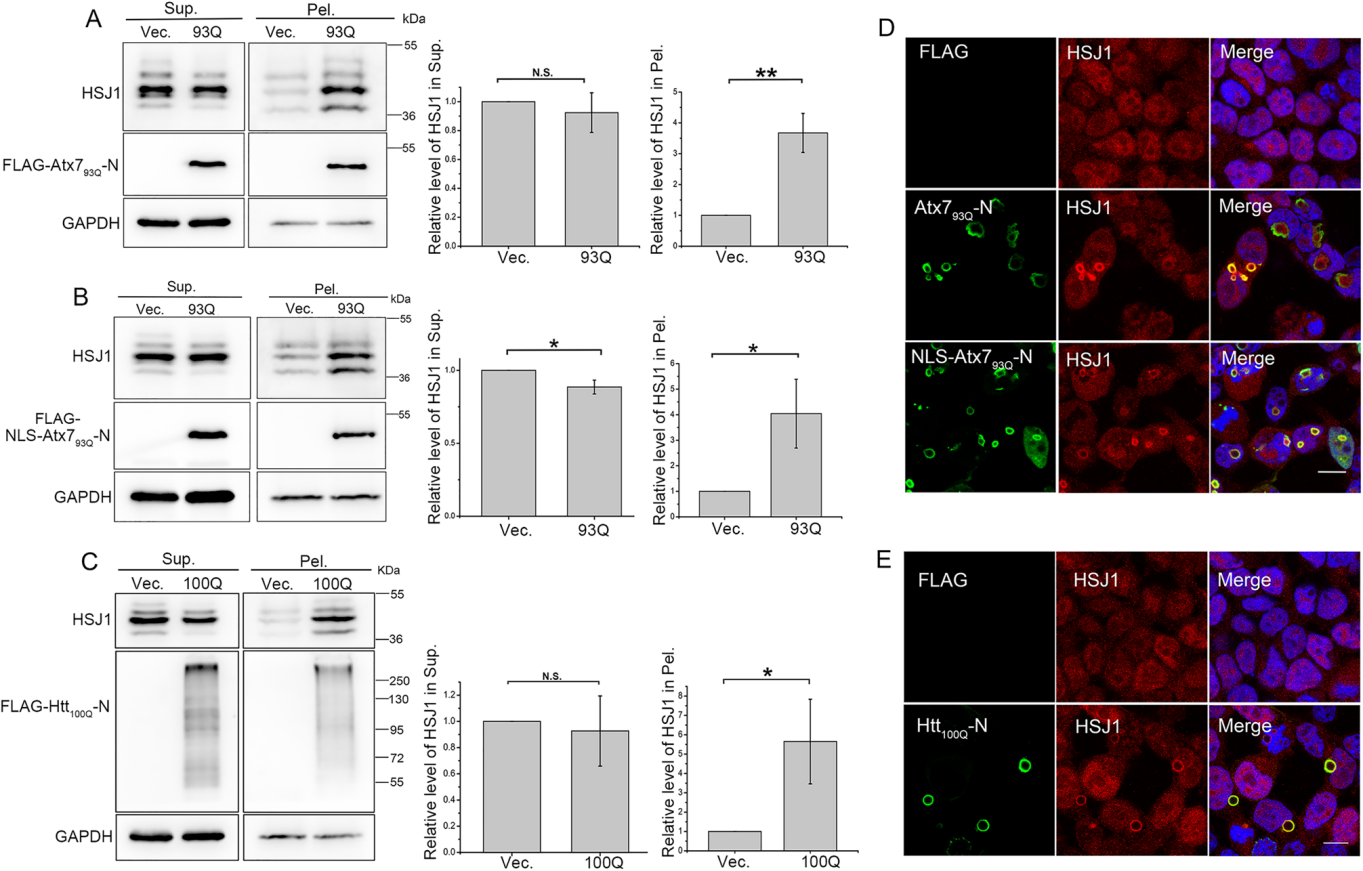
PQE Atx7 and Htt sequester endogenous HSJ1 into aggregates. (**A**) Sequestration of endogenous HSJ1 by Atx7_93Q_-N172. (**B**) As in (**A**), by NLS-Atx7_93Q_-N172. (**C**) As in (**A**), by Htt_100Q_-N90. HEK 293T cells were transfected with each indicated plasmid and the lysates were subjected to supernatant/pellet fractionation and Western blotting. Indicated proteins were detected by using anti-FLAG, anti-HSJ1 and anti-GAPDH antibodies. The three main bands indicate different isoforms of endogenous HSJ1. Sup., supernatant; Pel., pellet. Data are shown as Means ± SEM (n = 3). *p < 0.05; **p < 0.01; N.S., no significance. (**D**) Immunofluorescence imaging showing that Atx7_93Q_-N172 sequesters endogenous HSJ1 into inclusions. (**E**) As in (**D**), Htt_100Q_-N90. HEK 293T cells were transfected with indicated plasmids and then subjected to immunofluorescence imaging. PolyQ proteins were stained with anti-FLAG antibody (green), HSJ1 was stained with anti-HSJ1 antibody (red), and nuclei were stained with Hoechst (blue). Scale bar = 10 μm.

 As is the critical role of HSJ1a in modulating cellular Atx3 proteostasis^45^, we therefore inferred that the PQE proteins may disturb Atx3 proteostasis through interfering with the function of HSJ1. To address this issue, we investigated the effect of the PQE proteins on HSJ1 in cells. As shown (Supplemental Fig. 4), upon expression of Atx7_93Q_-N172 or NLS-Atx7_93Q_-N172, exogenous HSJ1a in the pellet fraction increased significantly, whereas it remained unchanged in the supernatant. We then examined the effects of these PQE Atx7 and Htt on the endogenous HSJ1, mainly HSJ1a and HSJ1b forms. The protein level of endogenous HSJ1 in pellet became much higher upon expression of Atx7_93Q_-N172 (Fig. 5A), NLS-Atx7_93Q_-N172 (Fig. 5B**)** or Htt_100Q_-N90 (Fig. 5C), as compared with that of the control vector. There is a slight decrease of the HSJ1 level in supernatant when over-expressing the nucleus-localized form (Fig. 5B), which was not observed in the cytoplasmic forms. Thus, HSJ1 may be sequestered into the aggregates by the PQE proteins. For further confirmation, we observed the cellular localizations of HSJ1 and the polyQ inclusions by confocal microscopy. The images showed that endogenous HSJ1 was well co-localized with the inclusions formed by these three polyQ proteins (Fig. 5D, E). Together, these data demonstrate that the PQE proteins can sequester HSJ1 into their aggregates or inclusions.

### Importance of the UIM domain of HSJ1 in sequestration by the PQE proteins

**Figure 6:**
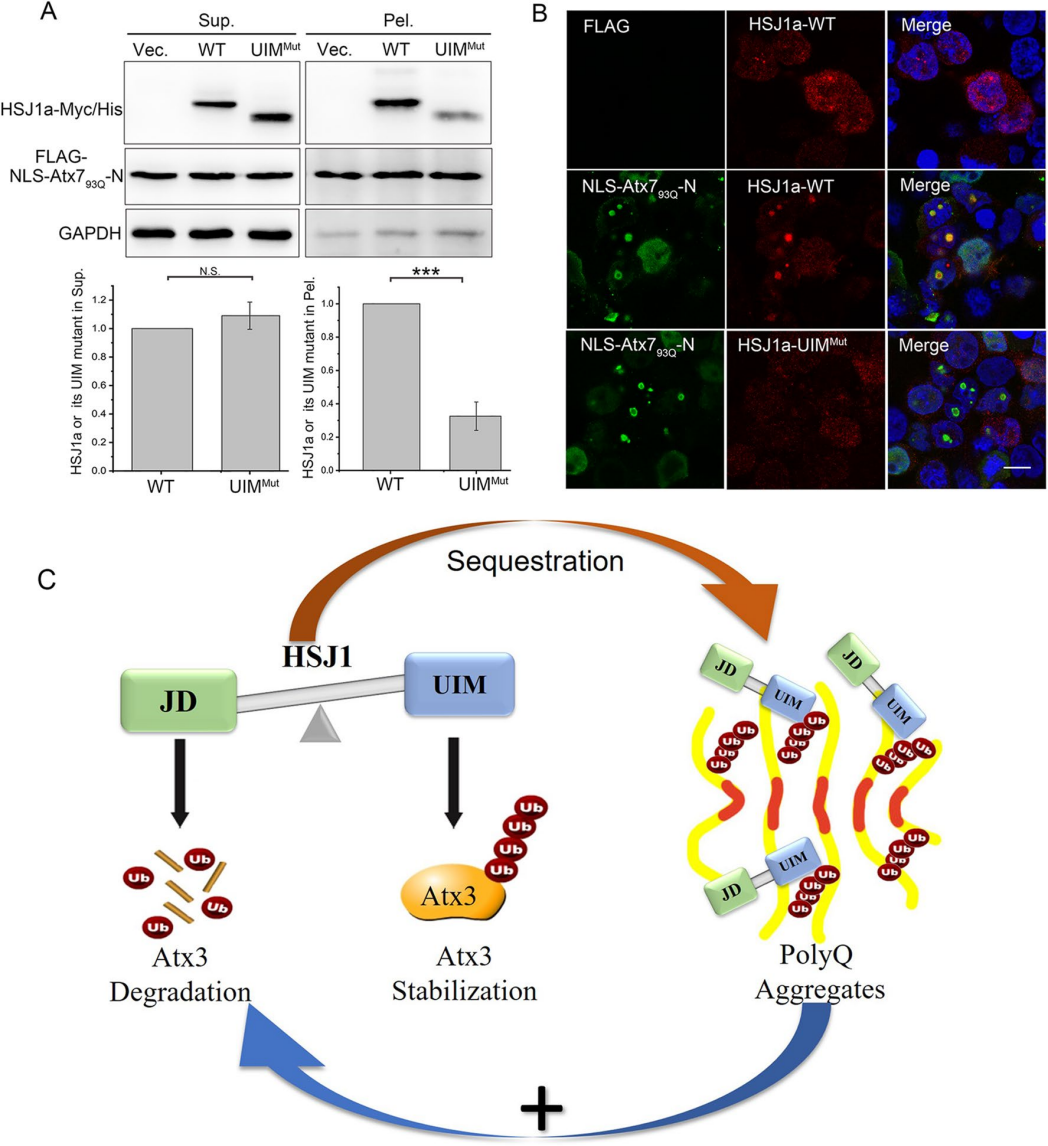
Sequestration of HSJ1 by the polyQ aggregates is mediated by the UIM domain of HSJ1. (**A**) UIM mutation suppresses the sequestration of HSJ1a into aggregates. HSJ1a-Myc/His or its UIM mutant was co-expressed with NLS-Atx7_93Q_-N172 in HEK 293T cells, the cell lysates were subjected to supernatant/pellet fractionation, then HSJ1a or its UIM mutant was detected by Western blotting. Sup., supernatant; Pel., pellet. Data are shown as Means ± SEM (n = 3). ***p < 0.001. N.S., no significance. (**B**) Sequestration of HSJ1a or its UIM mutant into polyQ inclusions as visualized by confocal microscopy. HSJ1a-Myc/His or its UIM mutant was co-expressed with NLS-Atx7_93Q_-N172 in HEK 293T cells, then the cells were subjected to immunofluorescence imaging. NLS-Atx7_93Q_-N172 were stained with anti-FLAG antibody (green), HSJ1a-Myc/His or its UIM mutant was stained with anti-Myc/His antibody, and nuclei were stained with Hoechst (blue). Scale bar = 10 μm. (**C**) Schematic representation of the impaired proteostasis of Atx3 caused by polyQ aggregates through sequestration of HSJ1. Under the normal conditions, HSJ1 balances the degradation and stabilization of Atx3 (brown) through coordinating the functions of its JD and UIM domains, and thus maintains the cellular proteostasis of Atx3. The PQE protein (yellow) forms insoluble aggregates via its polyQ tract (red bar), interacts with HSJ1 via its conjugated Ub molecules (red ball), and sequesters HSJ1 into the aggregates. The sequestration of HSJ1 may cause depletion or dysfunction of the co-chaperone and consequently impairs the cellular proteostasis of Atx3.

 According to our hypothesis, molecular interactions are the prerequisite for sequestration by protein aggregates^40,54^. To better understand the mechanism underlying the sequestration effect of PQE proteins on HSJ1, we constructed two domain-deleted mutants of HSJ1a, HSJ1a-ΔJD and HSJ1a-ΔUIM, and visualized their co-localization with the nuclear polyQ inclusions. As shown, full-length HSJ1a was localized to the inclusions formed by NLS-Atx7_93Q_-N172 in nuclei, while the JD-deleted mutant was also observed well co-localized with the polyQ inclusions in nuclei, but deletion of UIM disrupted their co-localization in the inclusions (Supplemental Fig. 5). It suggests that the UIM domain of HSJ1a may be critical for its being sequestered into the polyQ aggregates or inclusions. We then applied the HSJ1a-UIM^mut^ mutant and examined the sequestration efficiency by the nucleus-localized form of Atx7_93Q_-N172. The result showed that the sequestered protein level of the UIM mutant of HSJ1a was much lower than that of the WT (Fig. 6A). Consistently, as observed by confocal microscopy, the UIM mutation significantly alleviated the localization of HSJ1a into the polyQ inclusions formed by NLS-Atx7_93Q_-N172 in nuclei (Fig. 6B). Thus, the UIM domain of HSJ1 is important to be sequestered into the polyQ aggregates or inclusions, which implies the specific interaction of HSJ1 with the Ub moieties conjugated in the polyQ proteins^55^.

## Discussion

### The PQE protein aggregates disrupt cellular proteostasis of Atx3

 As the pathogenic protein of SCA3, PQE Atx3 has received quite a lot of attention from the researchers^43^. In the recent years, WT Atx3 has been found in the inclusion bodies or aggresomes formed by the misfolded CFTR mutant (CFTR-ΔF508)^41^, SOD1^42^ and even PQE Atx3 itself^17,44^. Thus, uncovering the link between Atx3 with those disease-related polyQ proteins is of great importance, but up to date, limited work has been done to elucidate this issue. As reported, WT Atx3 was passively sequestered into polyQ aggregates or inclusions by the co-aggregation of common polyQ sequences^17,44^, which may contribute to the reducing effect of the soluble or functionally available fraction of Atx3 in cell. We have revealed for the first time that the PQE proteins disrupt the protein balance of intracellular Atx3 through sequestering the co-chaperone HSJ1 into aggregates or inclusions. Specially, the PQE proteins promote the proteasomal degradation of endogenous Atx3 massively, leading to a remarkable reduction of the soluble Atx3 (Fig. 1). Besides, they can also enhance formation of the Atx3 aggregates accumulated in the inclusions. This accumulation of Atx3 aggregates, from our point of view, may be partially due to the increased misfolding and aggregation of Atx3 or attributed to the co-aggregation effect between the common and the expanded polyQ sequences. The severe damage of the proteostasis of intracellular Atx3 may further influence its normal function. Similar case has also been reported that the normal function of Atx3 in stabilization of CREB-binding protein (CBP) may be destroyed by mutant Htt, and consequently lead to dysregulation of the CBP-related gene expression^56^. It is likely that the loss-of-function effect of Atx3 resulted from disruption of the cellular proteostasis may account for one of the pathologies in Huntington’s disease.

### HSJ1 functions in modulating the cellular proteostasis of Atx3

 The HSJ1 protein is an essential player orchestrating the proteostasis in nerve cells^46,57^, for example, overexpression of HSJ1a is probable to reduce aggregation of mutant (PQE) Htt and enhance its solubility^58^. Mutations in HSJ1 are found to be closely related to the pathologies of diverse neurodegenerative disorders^59–61^. Work in our laboratory has identified HSJ1 as a regulator of both the WT and pathogenic Atx3 protein^45^, which further emphasizes the importance of HSJ1 in cellular proteostasis and proteinopathy. HSJ1 exhibits dual roles in regulating the degradation and stabilization of Atx3 through its JD and UIM domains, respectively. The JD and UIM domains of HSJ1 seem to delicately maintain the balance of the cellular Atx3 protein in a *Yin-Yang* manner. However, expression of the PQE proteins significantly destroys this internal balance, leading to the massive degradation of soluble Atx3. Overexpression of the full-length or JD-deleted HSJ1a rather than the UIM-mutated one can efficiently suppress the degradation of Atx3 and reverse its protein level, which confirms the role of the UIM domain of HSJ1 in maintaining the Atx3 stability^45^. Apart from the function in modulating protein stability, HSJ1 also exhibits a chaperoning activity by binding with HSP70 and promoting proteasomal degradation, which to some extent attenuates formation of the Atx3 aggregates. The anti-aggregation role of the HSP40 co-chaperones has also been documented in other literatures^62–64^, making it a potential candidate of drug targets for manipulating the amyloid related diseases.

### Sequestration of HSJ1 by the polyQ aggregates

 Accumulating evidence has shown that molecular chaperones are the common components accumulated in inclusion bodies^7,35^. As a member of the co-chaperone family, HSJ1 has also been observed in polyQ inclusions^36,45^. This study corroborates the previous finding that HSJ1 can be accumulated in the aggregates or inclusions through interacting with the PQE proteins (Fig. 5). According to our hijacking hypothesis raised previously^40^, HSJ1 could be sequestered into the polyQ aggregates or inclusions that may cause dysfunction of the co-chaperone. We propose that this sequestration effect partially contributes to the proteinopathies of polyQ proteins. Since the chaperone and co-chaperone components play a central effect in protein quality control (PQC), the excessive occupation or sequestration of those proteins may thus destroy the integrity and homeostasis of the intracellular proteome. For example, sequestration of Sis1p (an HSP40-like co-chaperone in yeast) into the polyQ aggregates results in inefficient degradation of the cytoplasmic misfolded proteins and disruption of their cellular proteostasis^39^. Our results demonstrate that HSJ1 could be sequestered into the polyQ aggregates through its UIM domain (Fig. 6A, B). Analogous to our previous finding that the PQE proteins sequester Ub adaptors into aggregates via conjugated Ub chains^24^, we speculate that the PQE protein fragments (Atx7_93Q_-N172 and Htt_100Q_-N90) that are prone to forming aggregates are highly ubiquitinated^55^, so that they are capable of binding with HSJ1 on its UIM domain. Considering the function of HSJ1 in maintaining the balance of cellular Atx3, the PQE proteins impair the cellular proteostasis of Atx3 by sequestering HSJ1 into aggregates and causing the functional depletion or dysfunction of HSJ1 through UIM-Ub interactions.

### The PQE proteins disturb the modulating balance of HSJ1 on endogenous Atx3 by the sequestration effect

 Our laboratory has proposed that protein aggregates sequester essential cellular components and thus cause the loss-of-function effect^40^, which may explain why protein aggregates are toxic to cells. Considering the importance of HSJ1 in maintaining the balance of cellular Atx3, we conclude that the PQE proteins impair cellular proteostasis of Atx3 through sequestering HSJ1 into aggregates and consequently causing functional loss of HSJ1 (Fig. 6C). Specifically, under normal conditions, the cellular proteostasis of Atx3 is sophisticatedly balanced by the co-chaperone HSJ1, in which the J domain mediates proteasomal degradation of Atx3 while the UIM domain stabilizes the ubiquitinated Atx3^45^. However, the PQE protein may experience ubiquitination that confers it to interact with the UIM domain of HSJ1; as a result, the cellular HSJ1 protein will be massively sequestered into the ubiquitinated polyQ aggregates. In this way, HSJ1 may be depleted by both sequestration into the insoluble polyQ aggregates and aberrant interactions with the Ub-conjugated protein monomers or oligomers. Depletion of the co-chaperone from its functionally available fraction will consequently break down the balance between degradation and stabilization of the Atx3 protein (Fig. 6C). Besides, this study also provides additional evidence in supporting the hijacking model that sequestration of cellular essential components by protein aggregates may account for the pathologies of aggregation-related neurodegenerative diseases.

## Materials and methods

### Plasmids, antibodies, and reagents

 The cDNAs for encoding Atx7_93Q_-N172 (residues 1–172) and Htt_100Q_-N90 (residues 1–90) were cloned into the FLAG-pcDNA3.1 vector as stored in our laboratory^45^. To construct a nucleus-localized expression plasmid, the DNA sequence for nuclear localization signal (NLS, PKKKRKV) was introduced right before that of Atx7_93Q_-N172. The cDNAs for encoding full-length HSJ1a (residues 1–274), HSJ1a-ΔJD (residues 91–274), HSJ1a-ΔUIM (residues 1–207) and HSJ1a-UIM^Mut^ (S219A/E222A/S262A/E265A) were cloned into the Myc/His-pcDNA3.0 vector^45^ (Supplemental Table 1). The antibody against FLAG was purchased from Sigma and anti-Atx3 was from Abclonal. The anti-HSJ1, anti-β-actin and anti-GAPDH antibodies were from Proteintech; anti-Myc and anti-LC3B were purchased from Cell Signaling (Supplemental Table 2). All secondary antibodies were from Jackson ImmunoResearch Laboratories. MG132 was purchased from Cell Signaling and chloroquine (CQ) diphosphate was from Sigma.

### Cell culture and transfection

 As described previously^25^, cells were cultured in DMEM (HyClone) supplemented with 10% fetal bovine serum (Gemini) and penicillin–streptomycin at 37 °C under a humidified atmosphere containing 5% CO_2_. Transfections of all the plasmids into HEK 293T cells were performed by using *PolyJet* reagent (SignaGen) following the manufacturer’s instructions.

### Supernatant/pellet fractionation

 HEK 293T cells were harvested about 48 h after transfection and lysed in 100 μL of a RIPA buffer (50 mM Tris–HCl, pH 7.5, 150 mM NaCl, 1 mM EDTA, 1% Nonidet P-40, and protease inhibitor mixture) on ice for 30 min, and then centrifuged at 16,200*g* for 15 min at 4 °C. The supernatant was added with 100 μL of 2× loading buffer (2% SDS), while the pellet was sufficiently washed with the RIPA buffer for three times at 4 °C before added with 40 μL of 4× loading buffer (4% SDS)^24^. Samples acquired through the above process were then subjected to SDS-PAGE and Western blotting.

### Extraction of total Atx3 protein

 The cultured cells were harvested about 48 h after transfection, and lysed with 100 μL of the RIPA buffer containing 8 M urea on ice for 30 min for dissolving all aggregated forms of Atx3. The lysates were then added with 100 μL of 2× loading buffer (2% SDS) that also contains 8 M urea. The protein samples acquired through the above method were then boiled to denature and subjected to SDS-PAGE and Western blotting.

### Western blotting analysis

 The protein samples of lysates or fractions were subjected to SDS-PAGE and transferred onto PVDF membranes (PerkinElmer). When needed, the blots had been cut prior to antibody hybridization. The indicated proteins were detected with specific primary and secondary antibodies and visualized by using an ECL detection kit (Thermo Scientific) as previously described^65^. For quantification, the integral grayscale values of protein bands were recorded by using *Scion Image* or *ImageJ* software. All the original un-processed gel images are provided in the Supplementary Information file.

### Immunofluorescence microscopy

 The method for preparing samples for confocal imaging was similar to that described previously^24^. HEK 293T cells grown on glass coverslips were transfected with indicated plasmids by *PolyJet* reagent. About 48 h after transfection, the cells were fixed with 4% paraformaldehyde for 15 min, permeabilized with 0.1% Triton X-100 and then blocked with the blocking solution (5% BSA and 10% FBS in a PBS buffer) for 1 h. All the above processes were performed at room temperature. Then the cells were incubated with the respective primary antibodies against FLAG (1:100) and Atx3 (1:100), HSJ1 (1:100) or Myc (1:100) overnight at 4 ^0^C. After washing with the PBS buffer for three times, the cells were labeled with FITC-conjugated anti-mouse antibody and TRITC-conjugated anti-rabbit antibody (1:100, Jackson ImmunoResearch Laboratories) for 1 h. The nuclei were stained with Hoechst (Sigma). All images were obtained on a Leica Microsystems TCS SP8 confocal microscope.

### Quantitative RT-PCR assay

Total RNAs from cultured HEK 293T cells were extracted with TRIzol (Life Technologies) according to the manufacturer’s protocol. The cDNAs were reversely transcribed with 4× Reverse Transcription Master Mix (EZBioscience) using 2-μg total RNA from each sample. Quantitative real-time PCR (qPCR) was performed using Hieff qPCR SYBR Green Master Mix (Yeasen Biotech) and analyzed on a LightCycler96 PCR system (Roche). The qPCR assay was carried out in a 20-μL reaction mixture (10 μL of SYBR, 2 μL of cDNA template and 0.5 mM each primer). GAPDH was chosen as the endogenous control for qPCR analysis. Three independent experiments were performed in this qPCR assay.

### Statistical analysis

 The data from at least three independent experiments were obtained from the gray values of specific protein bands in Western blots and normalized to that of the respective vector control. Then the relative levels for protein amounts were presented as Means ± SEM. Statistical analyses were performed with one-way ANOVA program by using *OriginPro* software. Differences were considered statistically significant at *p* < 0.05. In all experiments, the p values were labeled in the graphs with * (*p* < 0.05), ** (*p* < 0.01), *** (*p* < 0.001) and N.S. (no significance).

## Supplementary Information

Supplementary Informations.

## Abbreviations

Atx3: Ataxin-3
Atx7: Ataxin-7
CFTR: Cystic fibrosis transmembrane regulator
CREB: CAMP-response element binding protein
HSJ1: *Homo sapiens* J-domain protein
HSP40: Heat shock protein 40
Htt: Huntingtin
JD: J domain
NLS: Nuclear localization signal
polyQ: Polyglutamine
PQE: PolyQ expansion or polyQ-expanded
PQC: Protein quality control
RIPA: Radio-immune precipitation assay
RT-PCR: Reverse transcription-polymerase chain reaction
SCA3: Spinal cerebellar ataxia
SOD1: Copper-zinc superoxide dismutase 1
Ub: Ubiquitin
UIM: Ubiquitin-interacting motif
WT: Wild type
