# Supplementary material for: PolyQ-expanded proteins impair cellular proteostasis of ataxin-3 through sequestering the co-chaperone HSJ1 into aggregates
Source: Sci Rep. 2021 Apr 9;11:7815. doi: 10.1038/s41598-021-87382-w (PMC8035147; doi:10.1038/s41598-021-87382-w)
Supplement: Supplementary file 1 — Supplementary Informations. [file 41598_2021_87382_MOESM1_ESM.pdf]

Supplementary Information   Yue H.-W., et al.

PolyQ-expanded proteins impair cellular proteostasis of ataxin-3 through sequestering the co-chaperone HSJ1 into aggregates

Hong-Wei Yue[1,2], Jun-Ye Hong[1,2], Shu-Xian Zhang[1,2], Lei-Lei Jiang[1], Hong-Yu Hu[1,*]

[1] State Key Laboratory of Molecular Biology, Shanghai Institute of Biochemistry and Cell Biology, Center for Excellence in Molecular Cell Science, Chinese Academy of Sciences, Shanghai 200031, P. R. China.

[2] University of Chinese Academy of Sciences, Beijing 100049, P. R. China.

* Correspondence and requests for materials should be addressed to H.-Y. H. (email: hyhu@sibcb.ac.cn)

Content:

- Supplemental figures, Suppl. Fig. 1 - 5
- Supplemental tables, Suppl. Table 1 - 2
- Original uncropped blots, OriFig. 1 - 6, Ori. Suppl. Fig. 2 - 4

Suppl. Fig. 1

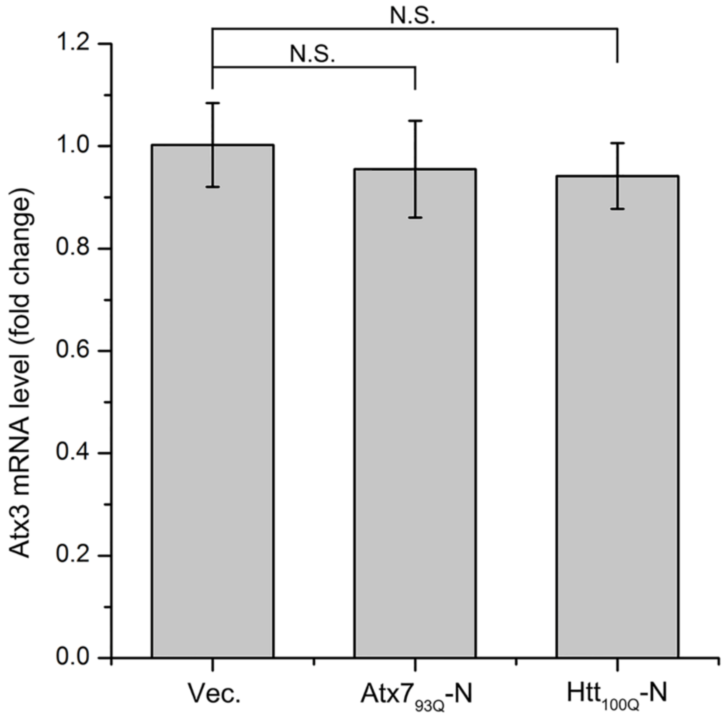

Suppl. Fig. 1. The mRNA levels of Atx3 upon overexpression of PQE protein fragments by RT-qPCR assay. HEK 293T cells were transfected with each indicated plasmid, and the mRNA level of Atx3 was determined by qPCR. The FLAG-tagged $Atx7_{93Q}$-N172 and Htt_{100Q}-N90 fragments were applied. N.S., no significance.

The primers used for the qPCR assay were as follows.

Primer	Sequence (5’-3’)
GAPDH-forward	GGAGCGAGATCCCTCCAAAAT
GAPDH-reverse	GGCTGTTGTCATACTTCTCATGG
Atx3-forward	ACAGACCTGGAACGAGTGTT
Atx3-reverse	CCATGTCAATTTCTTGGCGACT

Suppl. Fig. 2

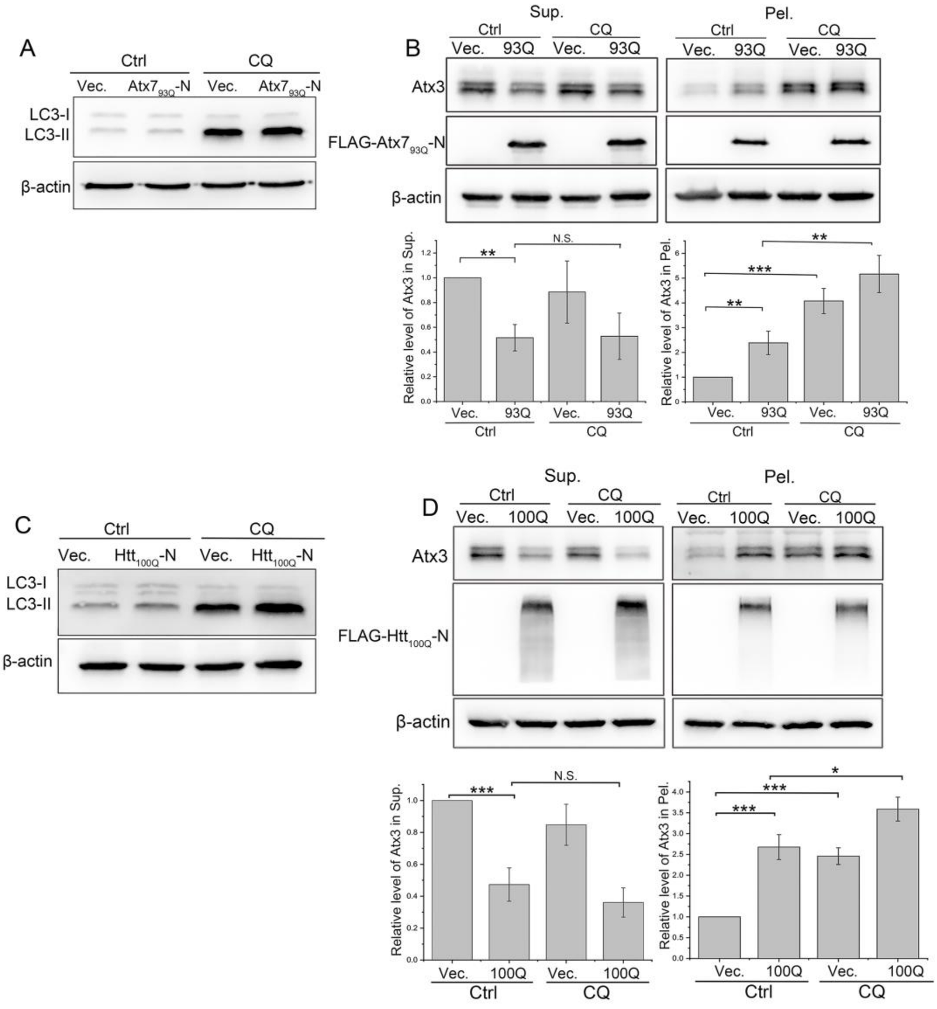

Suppl. Fig. 2. The autophagic pathway is not involved in the reduction of soluble Atx3 caused by the PQE proteins. (A) CQ treatment up-regulates the cellular LC3-II levels in the presence of Atx7$_{93Q}$-N172. (B) Inhibition of autophagy has no effect on the reduction of soluble Atx3 caused by Atx7$_{93Q}$-N172. (C) As in (A), in the presence of Htt$_{100Q}$-N90. (D) As in (B), by Htt$_{100Q}$-N90. HEK 293T cells were transfected with each indicated plasmid. Cells were treated with CQ (50 μM) for 12 hrs before harvest. The treatment with ddH$_2$O was set as a control (Ctrl). The cell lysates were subjected to supernatant/pellet fractionation, and then the protein levels of endogenous Atx3 were detected by Western blotting. LC3-II was detected by using anti-LC3B antibody. Sup., supernatant; Pel., pellet. Data are shown as Means ± SEM (n=3). *, $p<0.05$; **, $p<0.01$; ***, $p<0.001$; N.S., no significance.

Suppl. Fig. 3

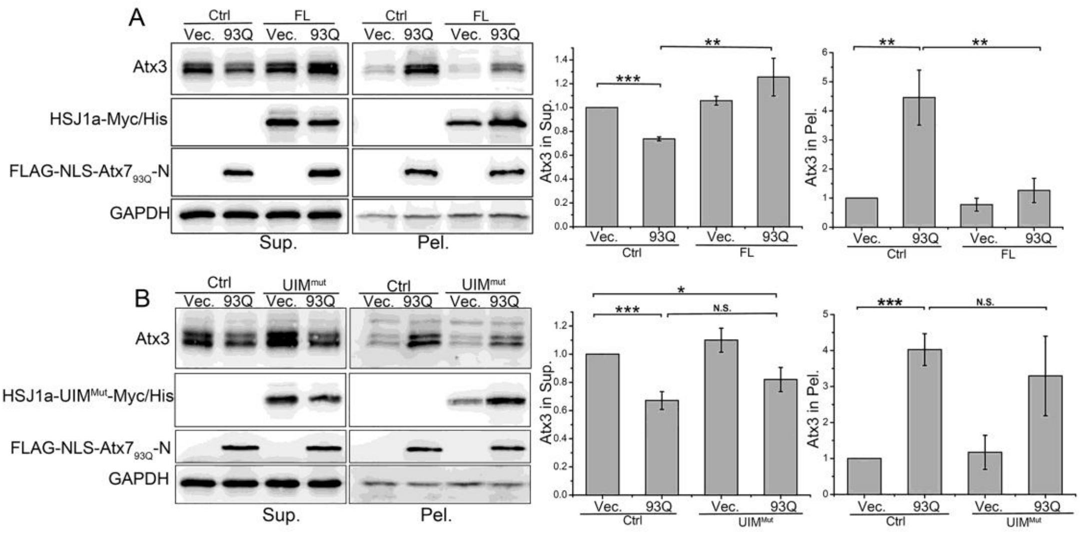

Suppl. Fig. 3. HSJ1a suppresses the degradation of Atx3 caused by NLS-Atx7$_{93Q}$-N172 through its UIM domain. (A) Overexpression of HSJ1a alleviates the decline of soluble Atx3 but still increases the aggregates caused by NLS-Atx7$_{93Q}$-N172. (B) Overexpression of HSJ1a-UIM^{Mut} fails to suppress the decline of soluble Atx3 caused by NLS-Atx7$_{93Q}$-N172. HSJ1a-Myc/His or HSJ1a-UIM^{Mut}-Myc/His was co-expressed with NLS-Atx7$_{93Q}$-N172 in HEK 293T cells. pcDNA3.1-Myc/His was set as a control. The cell lysates were subjected to supernatant/pellet fractionation, and then the endogenous Atx3 was detected by Western blotting. Sup., supernatant; Pel., pellet. Data are shown as Means ± SEM (n=3). *, $p<0.05$; **, $p<0.01$; ***, $p<0.001$; N.S., no significance.

Suppl. Fig. 4

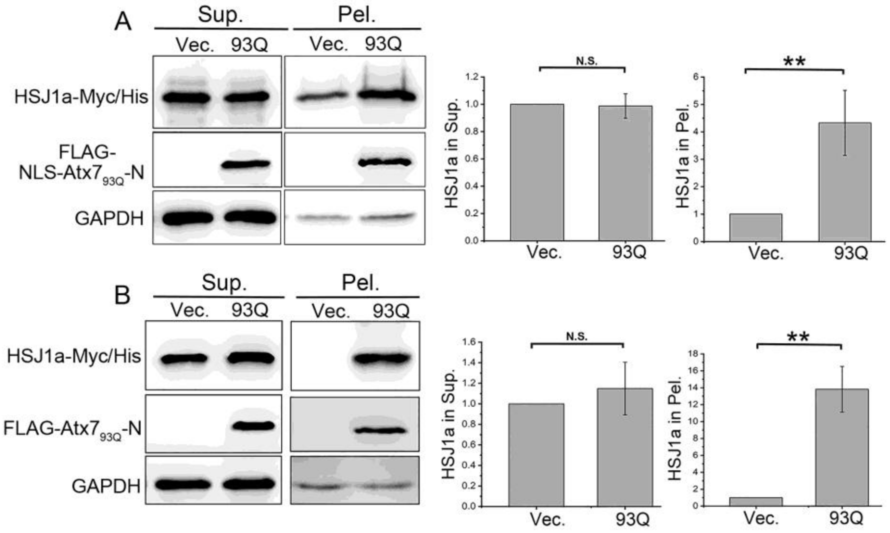

Suppl. Fig. 4. PQE Atx7 and Htt sequester exogenous HSJ1a into aggregates. (A) Sequestration of exogenous HSJ1a by nucleus-localized NLS-Atx7$_{93Q}$-N172. (B) Sequestration of exogenous HSJ1a by cytoplasm-localized Atx7$_{93Q}$-N172. HSJ1a-Myc/His was co-expressed with NLS-Atx7$_{93Q}$-N172 (A) or Atx7$_{93Q}$-N172 (B) in HEK 293T cells, the cell lysates were subjected to supernatant/pellet fractionation, and then exogenous HSJ1a was detected by Western blotting. Sup., supernatant; Pel., pellet. Data are shown as Means ± SEM (n=3). **, $p<0.01$; N.S., no significance.

Suppl. Fig. 5

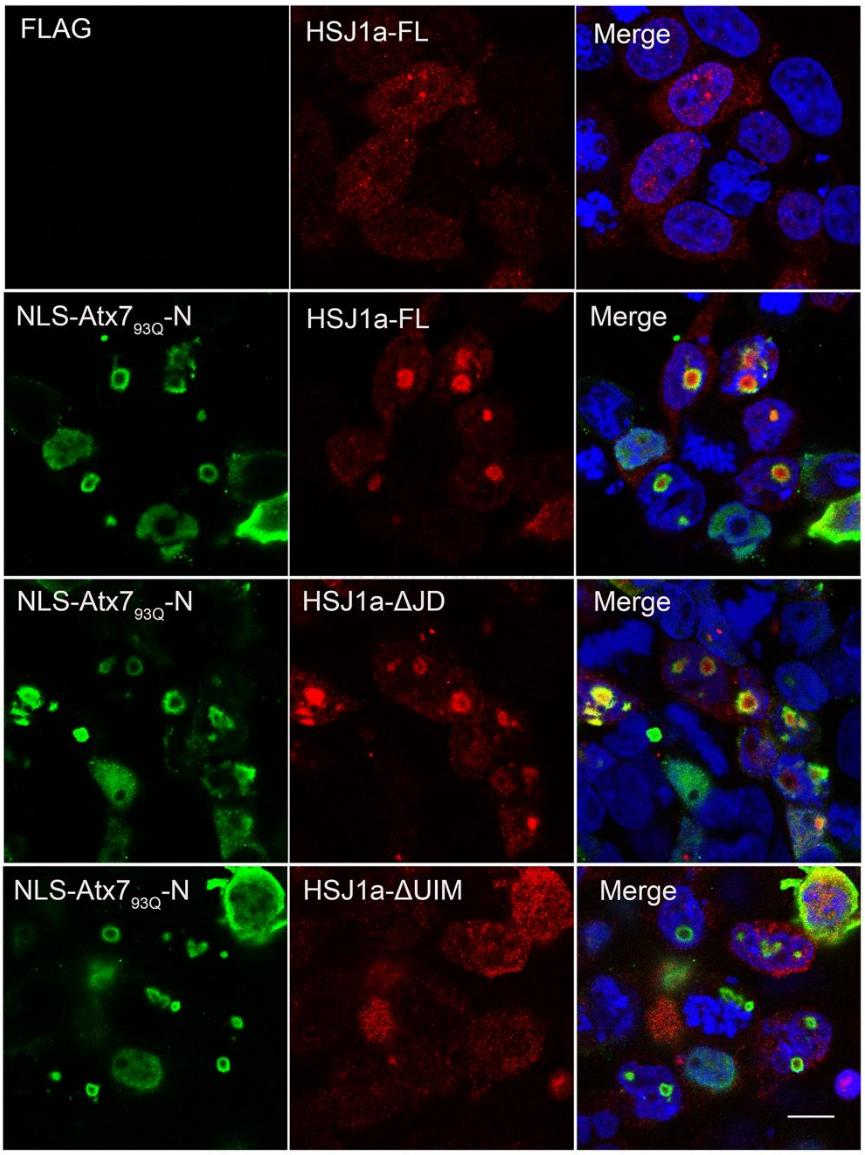

Suppl. Fig. 5. Confocal microscopic imaging showing that sequestration of HSJ1a into the inclusions is depending on its UIM domain. HSJ1a-Myc/His or its mutants (HSJ1a-ΔJD or HSJ1a-ΔUIM) was co-expressed with NLS-Atx7$_{93Q}$-N172 in HEK 293T cells, then the cells were subjected to immunofluorescence imaging. NLS-Atx7$_{93Q}$-N172 was stained with anti-FLAG antibody (green), HSJ1a-Myc/His or its mutants was stained with anti-Myc/His antibody, and nuclei were stained with Hoechst (blue). Scale bar = 10 μm.

Suppl. Table 1. List of the constructs applied in this study

**Constructs**	**Vectors**	**Restriction Enzyme sites**	**Additional**
FLAG-Atx7$_{93Q}$-N172	pcDNA3.1-FLAG	Bam HI/XhoI	Atx7, residues 1-172
FLAG-Htt$_{100Q}$-N90	pcDNA3.1-FLAG	Bam HI/XhoI	Htt, residues 1-90
FLAG-NLS-Atx7$_{93Q}$-N172	pcDNA3.1-FLAG	Bam HI/XhoI	NLS, PKKKRKV;<br>Atx7, residues 1-172
HSJ1a-Myc/His	pcDNA3.1-Myc/His	Bam HI/XhoI	HSJ1a, residues 1-274
HSJ1a-ΔJD-Myc/His	pcDNA3.1-Myc/His	Bam HI/XhoI	HSJ1a, residues 91-274
HSJ1a-ΔUIM-Myc/His	pcDNA3.1-Myc/His	Bam HI/XhoI	HSJ1a, residues 1-207
HSJ1a-UIM^{mut}-Myc/His	pcDNA3.1-Myc/His	BamHI/XhoI	HSJ1a, UIM mutant:<br>S219A/E222A/S262A/E265A

Suppl. Table 2. List of the antibodies used in this study

**Proteins**	**Antibodies**	**Source**	**Identifier**
FLAG-Atx7$_{93Q}$-N172	Anti-FLAG	Sigma-Aldrich	F7425-2MG
FLAG-Htt$_{100Q}$-N90	Anti-FLAG	Sigma-Aldrich	F7425-2MG
FLAG-NLS-Atx7$_{93Q}$-N172	Anti-FLAG	Sigma-Aldrich	F7425-2MG
HSJ1a-Myc/His	Anti-Myc	Cell Signaling	2272S
HSJ1a-ΔJD-Myc/His	Anti-Myc	Cell Signaling	2272S
HSJ1a-UIM^{mut}-Myc/His	Anti-Myc	Cell Signaling	2272S
ataxin-3	Anti-ataxin-3	Abclonal	A1243
HSJ1 (DNAJB2)	Anti-DNAJB2	Proteintech	10838-1-AP
LC3B	Anti-LC3B	Cell Signaling	3868S
GAPDH	Anti-GAPDH	Proteintech	60004-1-Ig
β-actin	Anti-β-actin	Proteintech	6008-1-Ig

Original uncropped blots

Ori. Fig. 1

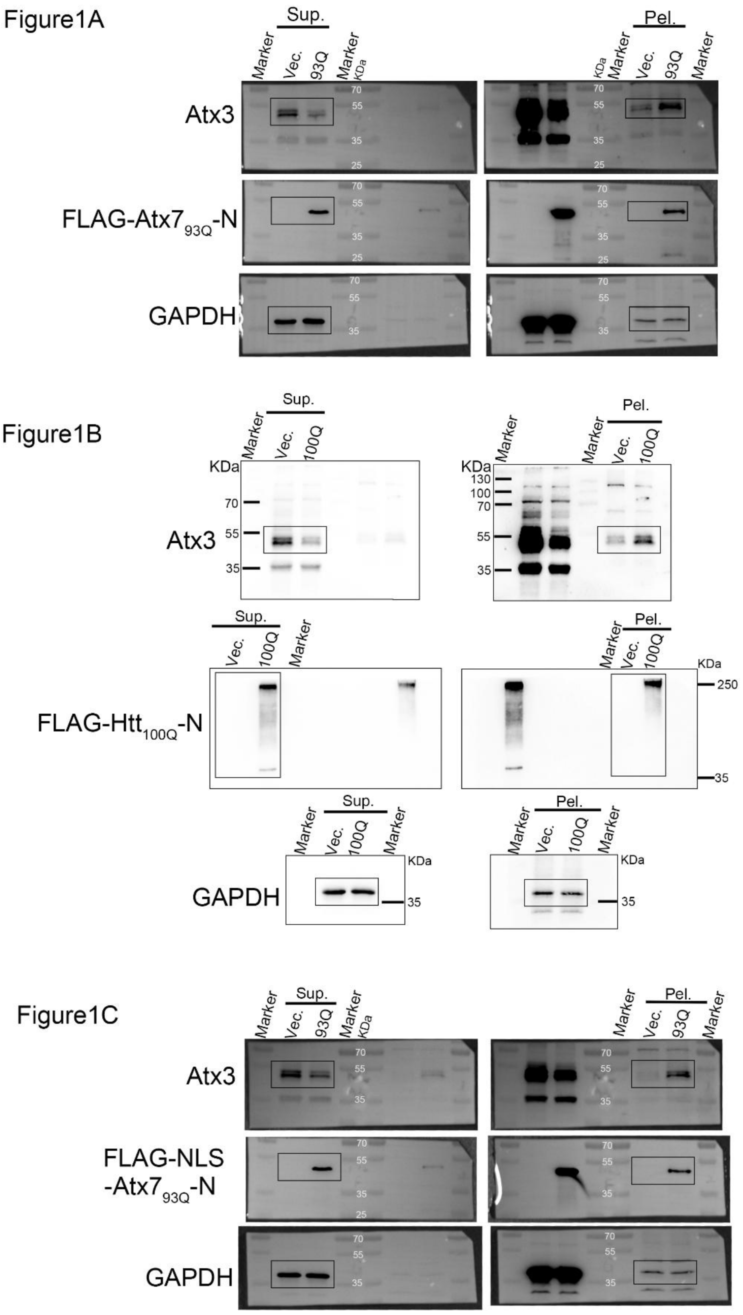

Fig. 1. PolyQ-expanded Atx7 and Htt reduce the soluble fraction of intracellular Atx3 but increase the insoluble. A, Effects of cytoplasm-localized Atx7$_{93Q}$-N172 on the supernatant and pellet fractions of Atx3. B, As in A, cytoplasm-localized Htt$_{100Q}$-N90. C, As in A, nucleus-localized NLS-Atx7$_{93Q}$-N172. HEK 293T cells were transfected with each indicated plasmid and the cell lysates were subjected to supernatant/pellet fractionation and Western blotting analysis. Indicated proteins were detected by using anti-FLAG, anti-Atx3 and anti-GAPDH antibodies. The two main bands indicate different endogenous isoforms of Atx3, Atx3-I and Atx3-II. Sup., supernatant; Pel., pellet. The blots had been cut prior to antibody hybridization. The protein bands shown in our article were marked in the black boxes.

Ori. Fig. 2

Figure2A

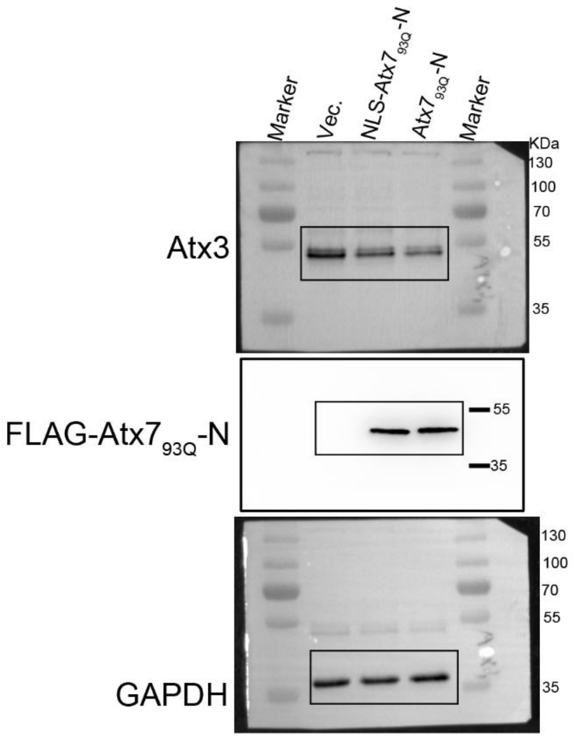

Figure2B

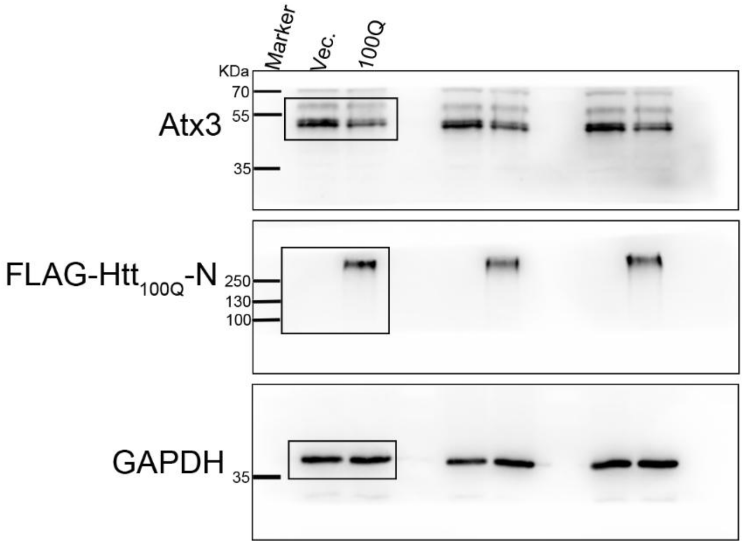

Fig. 2. PolyQ-expanded Atx7 and Htt reduce the overall protein level of intracellular Atx3. A, Effect of $Atx7_{93Q}$-N172 or NLS-$Atx7_{93Q}$-N172 on the overall level of Atx3. B, Effect of Htt_{100Q}-N90 on the overall level of Atx3. HEK 293T cells were transfected with each indicated plasmid and the total protein samples were prepared and subjected to Western blotting. Indicated proteins were detected by using anti-FLAG, anti-Atx3 and anti-GAPDH antibodies. The blots had been cut prior to antibody hybridization. The protein bands shown in our article were marked in the black boxes.

Ori. Fig. 3

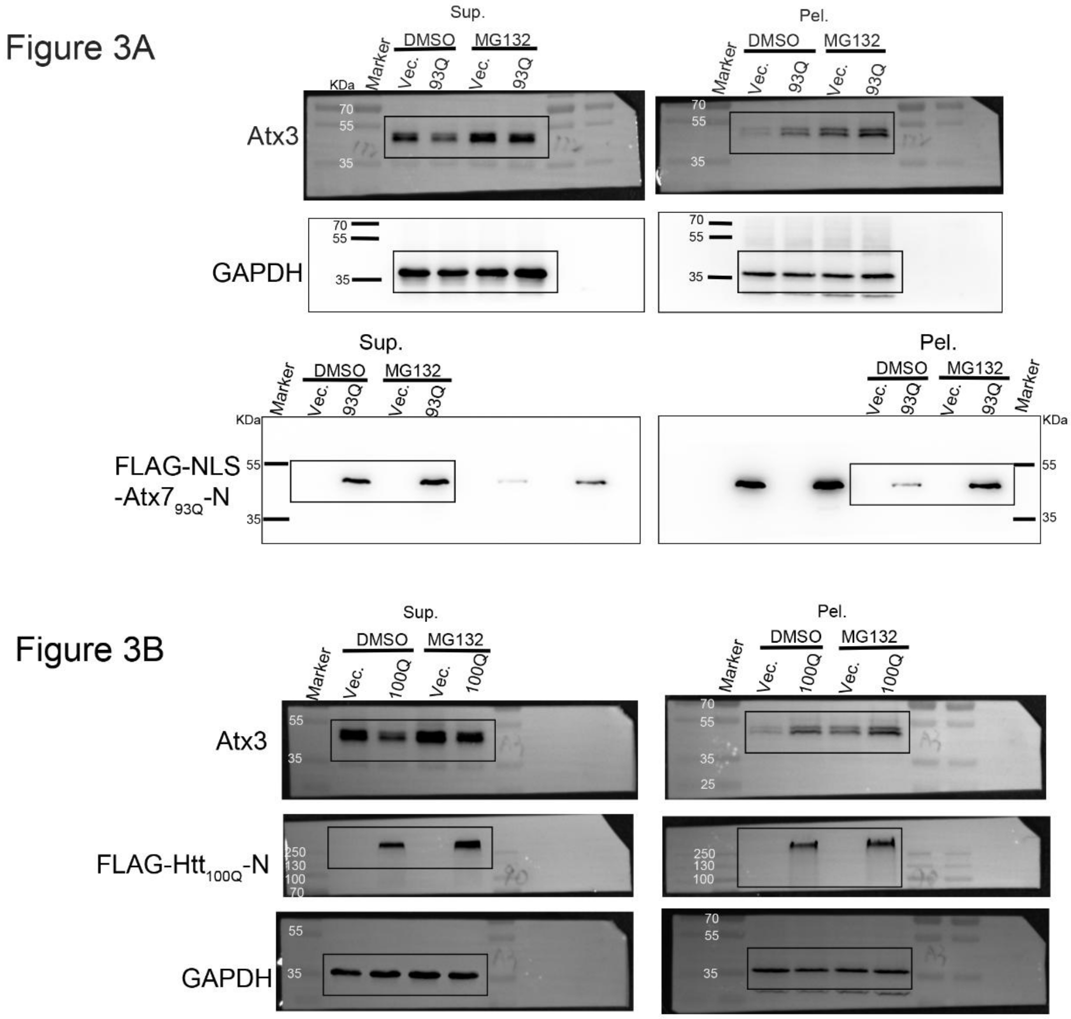

Fig. 3. PolyQ-expanded Atx7 and Htt cause reduction of soluble Atx3 through promoting proteasomal degradation. A & B, Proteasome inhibition suppresses the decline of soluble Atx3 caused by NLS-Atx7$_{93Q}$-N172 (A) or Htt$_{100Q}$-N90. HEK 293T cells were transfected with each indicated plasmid. Cells were treated with MG132 (10 μM) for 12 hrs before harvested. DMSO was set as a control. The cell lysates were subjected to supernatant/pellet fractionation and Western blotting. Indicated proteins were detected by using anti-FLAG, anti-Atx3 and anti-GAPDH antibodies. Sup., supernatant; Pel., pellet. The blots had been cut prior to antibody hybridization. The protein bands shown in our article were marked in the black boxes.

Ori. Fig. 4

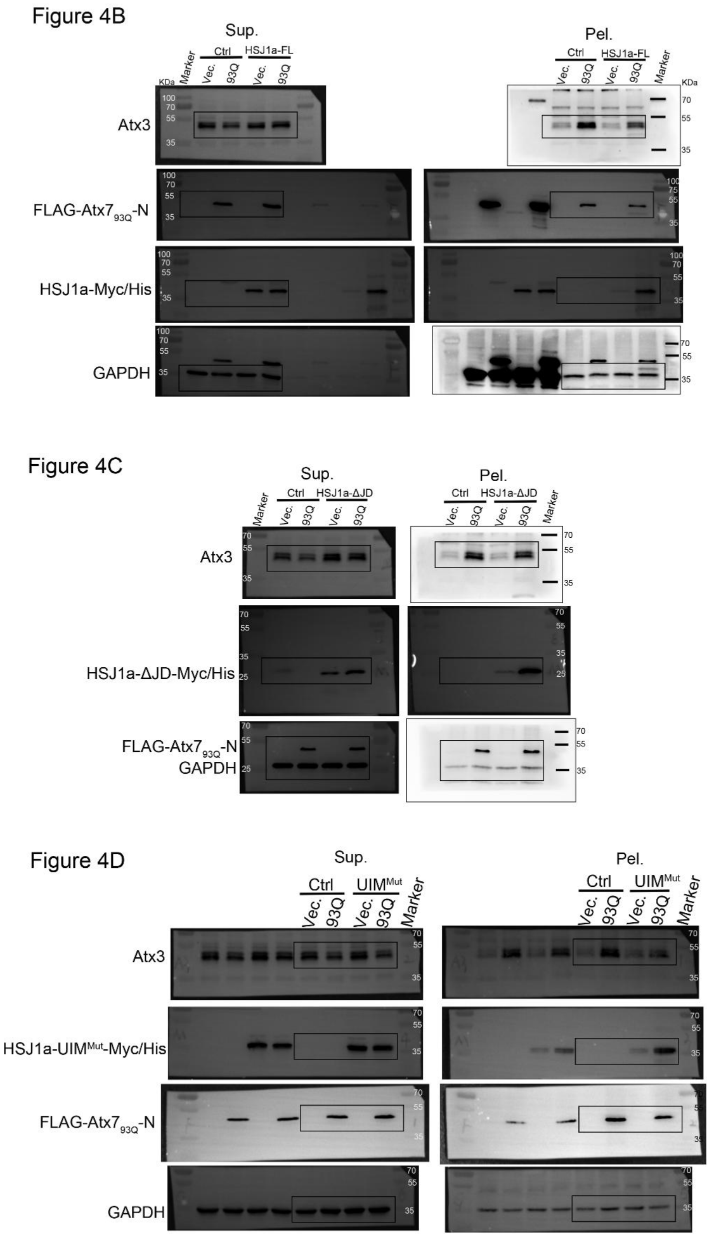

Fig. 4. HSJ1a suppresses the degradation of Atx3 through its UIM domain. B, Over-expression of HSJ1a alleviates the decline of soluble Atx3 but still increases the aggregates caused by Atx7$_{93Q}$-N172. C, As in B, HSJ1a-ΔJD. D, Over-expression of HSJ1a-UIM^{Mut} fails to suppress the decline of soluble Atx3 caused by Atx7$_{93Q}$-N172. HSJ1a-Myc/His or its mutants (HSJ1a-ΔJD and HSJ1a-UIM^{Mut}) was co-expressed with Atx7$_{93Q}$-N172 in HEK 293T cells. The pcDNA3.1-Myc/His plasmid was set as a control (Ctrl). The cell lysates were subjected to supernatant/pellet fractionation and Western blotting. Indicated proteins were detected by using anti-FLAG, anti-Myc, anti-Atx3 and anti-GAPDH antibodies. Sup., supernatant; Pel., pellet. The blots had been cut prior to antibody hybridization. The protein bands shown in our article were marked in the black boxes.

Ori. Fig. 5

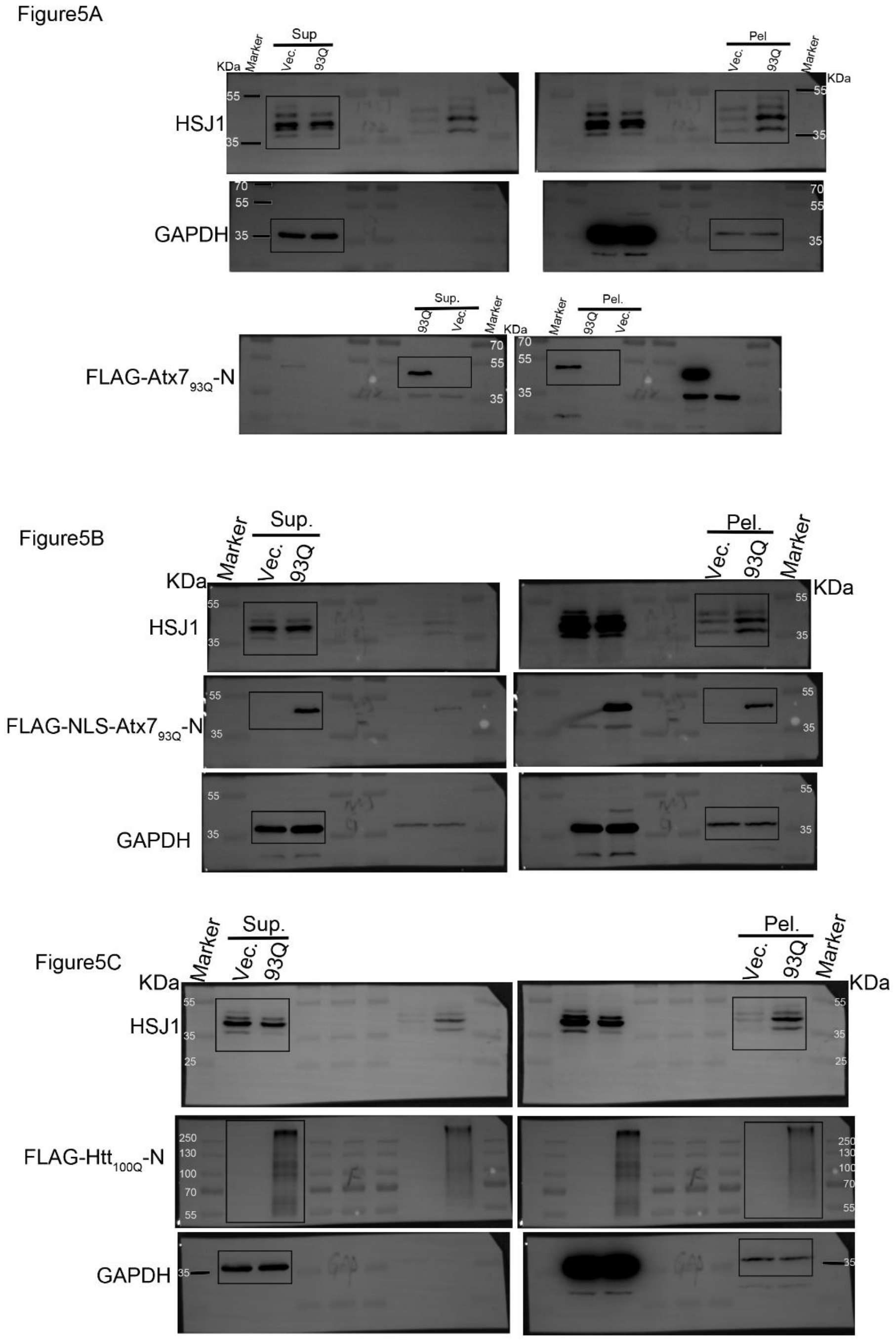

Note: In Fig. 5A, the gel for FLAG-Atx7$_{93Q}$-N172 was obtained from the same sample of lysates as the other two, but the lane order was reversed inconsciently during sample loading.

Fig. 5. PolyQ-expanded Atx7 and Htt sequester endogenous HSJ1 into aggregates. A, B & C, Sequestration of endogenous HSJ1 by Atx7$_{93Q}$-N172 (A), NLS-Atx7$_{93Q}$-N172 (B) or Htt$_{100Q}$-N90 (C). HEK 293T cells were transfected with each indicated plasmid and the lysates were subjected to supernatant/pellet fractionation and Western blotting. Indicated proteins were detected by using anti-FLAG, anti-HSJ1 and anti-GAPDH antibodies. The three main bands indicate different endogenous isoforms of HSJ1. Sup., supernatant; Pel., pellet. The blots had been cut prior to antibody hybridization. The protein bands shown in our article were marked in the black boxes.

Ori. Fig. 6

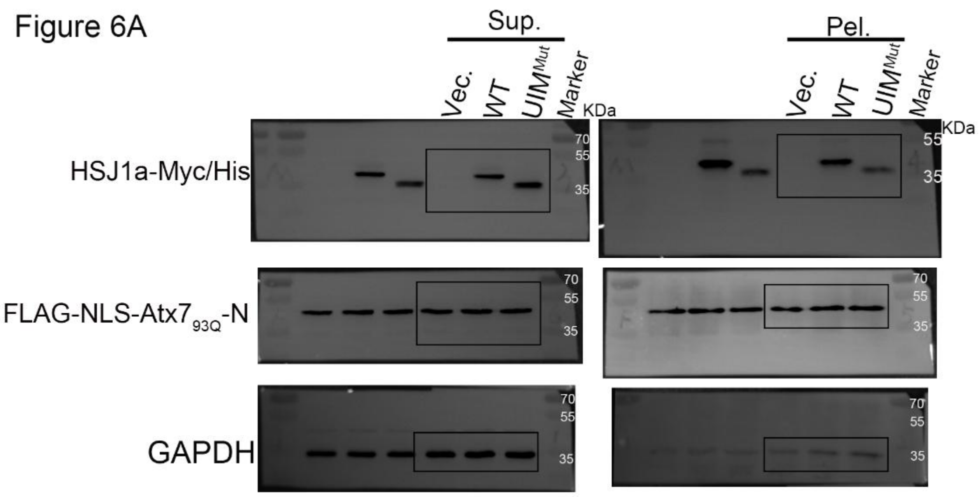

Fig. 6. Sequestration of HSJ1 by the polyQ aggregates is mediated by the UIM domain of HSJ1. A, UIM mutation suppresses the sequestration of HSJ1a into aggregates. HSJ1a-Myc/His or its UIM mutant was co-expressed with NLS-Atx7$_{93Q}$-N172 in HEK 293T cells, the cell lysates were subjected to supernatant/pellet fractionation, then HSJ1a or its UIM mutant was detected by Western blotting. Sup., supernatant; Pel., pellet. The blots had been cut prior to antibody hybridization. The protein bands shown in our article were marked in the black boxes.

Ori. Suppl. Fig. 2

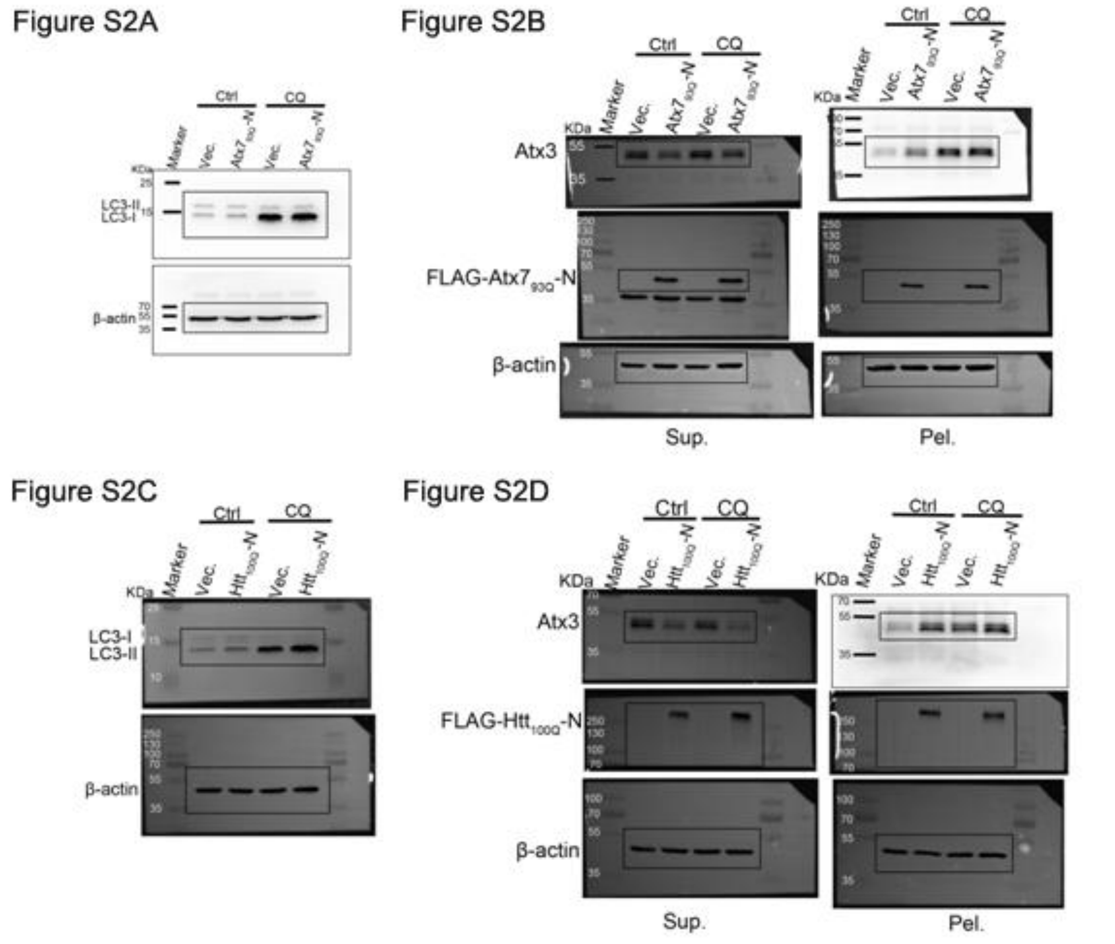

Suppl. Fig. 2. The autophagic pathway is not involved in polyQ-expanded protein-caused decline of soluble Atx3. A, CQ treatment up-regulates cellular LC3-II. B & D, Autophagy inhibition could not suppress the decline of soluble Atx3 caused by Atx7$_{93Q}$-N172 or Htt$_{100Q}$-N90. HEK 293T cells were transfected with each indicated plasmid. Cells were treated with CQ (50 μM) for 12 hrs before harvested. The ddH$_2$O treated groups were set as controls. The cell lysates were subjected to supernatant/pellet fractionation, then the endogenous LC3-II (A, C) or Atx3 (B, D) was detected by Western blotting. Sup., supernatant; Pel., pellet. The blots had been cut prior to antibody hybridization. The protein bands shown in our article were marked in the black boxes.

Ori. Suppl. Fig. 3

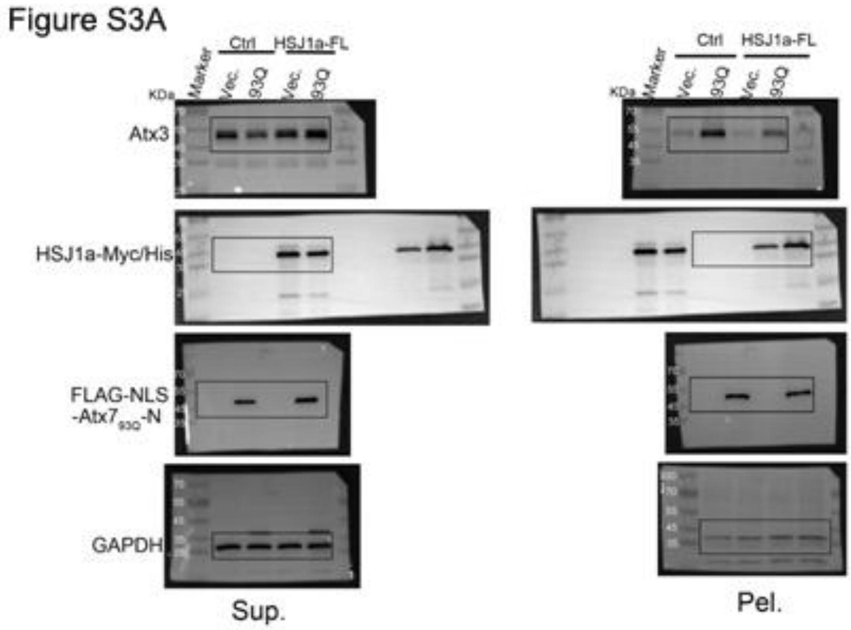

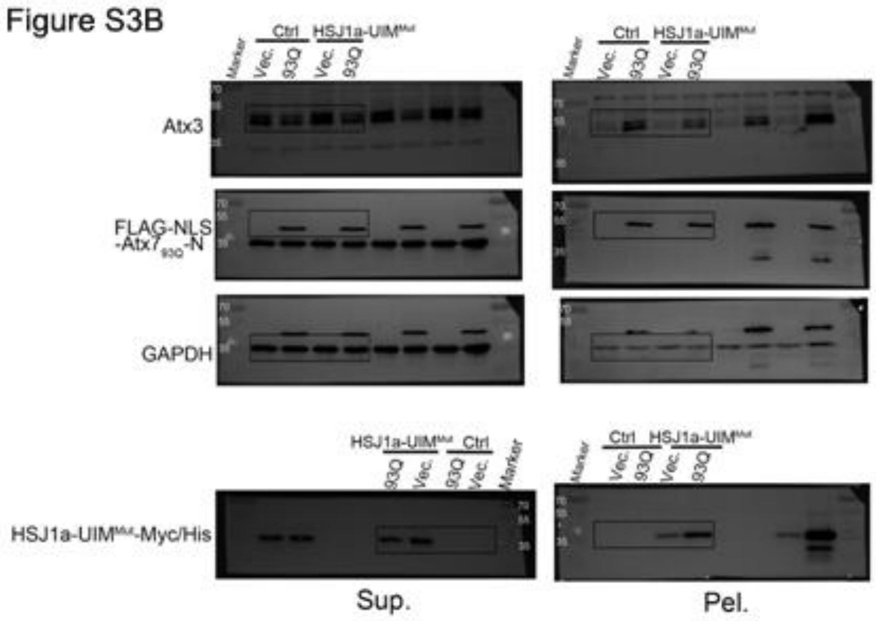

Note: In Suppl. Fig. 3B, the gel for HSJ1a-UIM^{Mut}-Myc/His in the supernatant was obtained from the same sample of lysates as the other three, but the lane order was reversed inconsciently during transferring.

Suppl. Fig. 3. HSJ1a supper sses the degradation of Atx3 caused by NLS-Atx7$_{93Q}$-N172 through the UIM domain. A, Over-expressing HSJ1a can suppress the decline of soluble of Atx3 and the increase of aggregated Atx3 caused by NLS-Atx7$_{93Q}$-N172. B, Over-expressing HSJ1a-UIM^{Mut} fails to suppress the decline of soluble of Atx3 caused by NLS-Atx7$_{93Q}$-N172. HSJ1a-Myc/His or HSJ1a-UIM^{Mut}-Myc/His was co-expressed with NLS-Atx7$_{93Q}$-N172 in HEK 293T cells. In the control (Ctrl) group, pcDNA3.1-Myc/His was co-expressed with NLS-Atx7$_{93Q}$-N172. The cell lysates were subjected to supernatant/pellet fractionation; then the endogenous Atx3 was detected by Western blotting. Sup., supernatant; Pel., pellet. The blots had been cut prior to antibody hybridization. The protein bands shown in our article were marked in the black boxes.

Ori. Suppl. Fig. 4

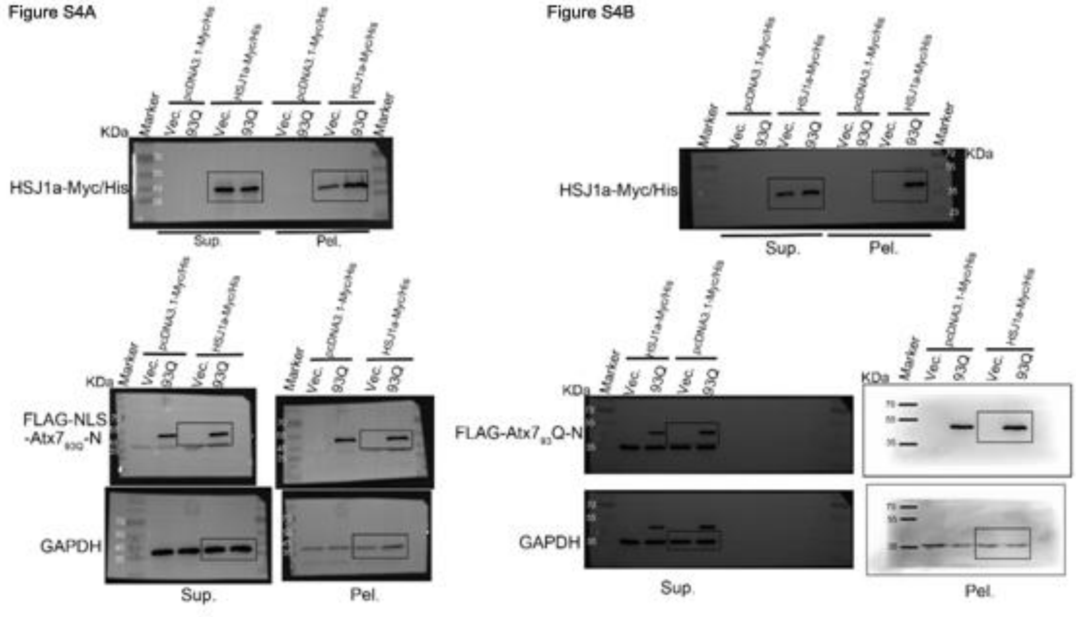

Suppl. Fig. 4. PolyQ-expanded proteins sequester exogenous HSJ1a into aggregates. A, NLS-Atx7_{93Q}-N172 sequesters exogenous HSJ1a into aggregates. B, Atx7_{93Q}-N172 sequesters exogenous HSJ1a into aggregates. HSJ1a-Myc/His was co-expressed with NLS-Atx7_{93Q}-N172 or Atx7_{93Q}-N172 in HEK 293T cells, the cell lysates were subjected to supernatant/pellet fractionation; then the exogenous HSJ1a was detected by Western blotting. Sup., supernatant; Pel., pellet. The blots had been cut prior to antibody hybridization. The protein bands shown in our article were marked in the black boxes.
